# Sequence-directed covalent protein–RNA linkages in a single step using engineered HUH-tags

**DOI:** 10.1093/nar/gkag517

**Published:** 2026-06-03

**Authors:** Adam T Smiley, Calvin J Thoma, Natalia S Babilonia-Díaz, August J Krueger, Andrew C D Lemmex, Aspen J Hughes, Matthew R Pawlak, Kassidy J Tompkins, Robert P Connacher, Hideki Aihara, Wendy R Gordon

**Affiliations:** Department of Biochemistry, Molecular Biology, and Biophysics, University of Minnesota, Minneapolis, MN 55455, United States; Department of Biochemistry, Molecular Biology, and Biophysics, University of Minnesota, Minneapolis, MN 55455, United States; Department of Biochemistry, Molecular Biology, and Biophysics, University of Minnesota, Minneapolis, MN 55455, United States; Department of Biochemistry, Molecular Biology, and Biophysics, University of Minnesota, Minneapolis, MN 55455, United States; Department of Biochemistry, Molecular Biology, and Biophysics, University of Minnesota, Minneapolis, MN 55455, United States; Department of Biochemistry, Molecular Biology, and Biophysics, University of Minnesota, Minneapolis, MN 55455, United States; Department of Biochemistry, Molecular Biology, and Biophysics, University of Minnesota, Minneapolis, MN 55455, United States; Department of Biochemistry, Molecular Biology, and Biophysics, University of Minnesota, Minneapolis, MN 55455, United States; Department of Biochemistry, Molecular Biology, and Biophysics, University of Minnesota, Minneapolis, MN 55455, United States; Department of Biochemistry, Molecular Biology, and Biophysics, University of Minnesota, Minneapolis, MN 55455, United States; Department of Biochemistry, Molecular Biology, and Biophysics, University of Minnesota, Minneapolis, MN 55455, United States

## Abstract

Replication-initiating HUH-endonucleases (reps) are enzymes that form covalent bonds with single-stranded DNA (ssDNA) in a sequence-specific manner to initiate rolling circle replication in plasmids and viruses. These nucleases have been co-opted for use in biotechnology as sequence-directed protein–ssDNA bioconjugation fusion partners dubbed ‘HUH-tags’. Here, we describe the engineering and *in vitro* characterization of a series of laboratory evolved HUH-tag derivatives of PCV2 called E1 and E2. E2 is capable of forming robust covalent bonds with unmodified RNA substrates in a sequence-specific manner. We show that promiscuous rep–RNA interaction can be enhanced through directed evolution from nearly undetectable levels in wildtype enzymes to robust reactivity in final engineered iterations. Subsequent *in vitro* characterization revealed that engineered enzymes have dramatically increased activity on both cognate ssDNA and noncognate ssRNA substrates. We benchmark our engineered proteins against an engineered variant called rHUH described in recent work from another laboratory pursuing the same function, and we perform an extensive analysis of sequence specificity across a range of metal ion concentrations. Together, these results establish a new class of RNA-reactive HUH-tags that expand the biochemical repertoire of this enzyme family and provide a promising platform for site-specific protein–RNA covalent bioconjugation. This technology has the potential to unlock diverse new applications in biotechnology and molecular engineering.

## Graphical Abstract

**Graphical Abstract:**
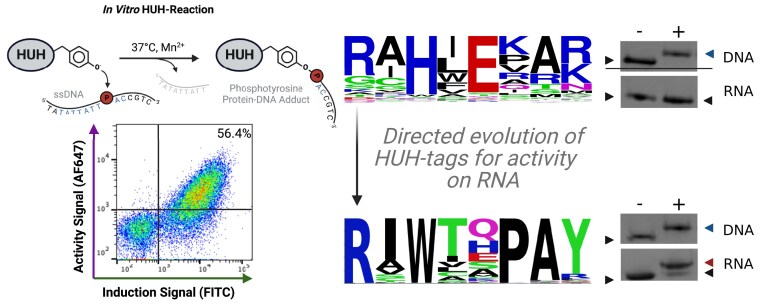

## Introduction

Synthetic protein–RNA interaction has proven itself to be an indispensable utility in basic science and biotechnology alike. One prominent example is the MS2 bacteriophage coat protein, which binds a cognate RNA hairpin with high specificity and affinity [1]. This system has been widely deployed in a diversity of applications, ranging from Cas9-mediated genome visualization [2], to the induction of trans-splicing in coordination with Cas13 [3], and even in the design of nanocages to protect RNAs in synthetic cell-to-cell mRNA delivery systems [4]. While affinity-based protein–RNA interactions have been employed extensively, covalent linkage between proteins and RNA offers distinct advantages, such as irreversible binding and enhanced stability through direct linkage of an exposed end, protecting the molecule from exonuclease degradation. However, covalent linkage also presents unique challenges as it often requires synthetic methods that rely on the chemical modification of surface exposed residues in order to facilitate the formation of a stable protein–RNA bond, which could inhibit the labeled protein’s biological function [5–8].

Enzymatic bioconjugation techniques have the potential to mitigate the negative aspects of synthetic methods by providing a more straightforward and efficient means by which to achieve covalent linkage between proteins of interest and RNA sequences of interest. The SNAP- [9], CLIP- [10], and HALO-tag [11] systems have emerged as a highly versatile suite of bioconjugation tags that can agnostically append to a molecular substrate so long as it contains their specific chemical moiety, making them promising candidates for mediating protein–RNA bioconjugation. Indeed, SNAP-tags have already been successfully deployed as fusions to adenosine deaminases acting on RNA (ADAR) enzymes to mediate covalent fixation of a guiding RNA to enable site-specific RNA editing [12]. Further, SNAP-tags have also been employed in a more generalizable strategy to mediate designer protein–RNA covalent interaction more broadly [13]. While these examples demonstrate the potential of enzymatic bioconjugation for protein–RNA linkage, the requirement for chemical synthesis to enable SNAP-tag based strategies somewhat limits their broad adoption in these applications. This limitation has prompted the exploration of alternative enzymatic strategies that could enable more cost-effective and accessible protein–RNA bioconjugation. One promising avenue lies in the repurposing of naturally occurring enzymes that form covalent bonds with nucleic acids as part of their native function.

The HUH-endonuclease superfamily comprises a diverse group of sequence-specific nucleases that interact with single-stranded DNA (ssDNA) and share a conserved HUH motif, characterized by a pair of metal-coordinating histidines (H) separated by a bulky hydrophobic residue (U) [14, 15]. This superfamily is comprised of replication initiator proteins (reps) involved in rolling-circle replication, relaxases involved in plasmid conjugation, and a diversity of DNA transposases ranging from the prokaryotic insertion sequence transposase families to the eukaryotic Helitron rolling-circle transposases and the newly discovered Replitron transposases [14–21].

**Figure 1.**
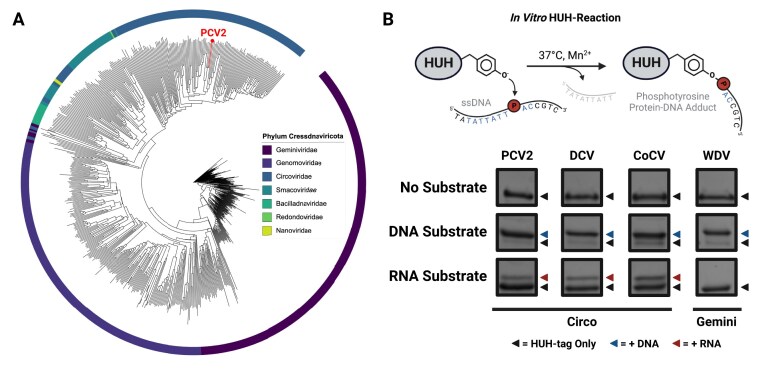
Discovering Promiscuous Interaction with RNA Substrates. (**A**) A phylogenetic tree of representative rep proteins from phylum *Cressdnaviricota* colored by viral family of origin for each of its seven members with the branch representing PCV2 specifically highlighted in red. (**B**) Graphical schematic of an *in vitro* HUH-tag mediated ssDNA-labeling reaction scheme above representative sodium dodecyl sulphate–polyacrylamide gel electrophoresis (SDS–PAGE) gels showing demonstrating the specified HUH-tag’s ability to covalently link to either DNA (top) or RNA (bottom) substrates as indicated by an increase in molecular weight. These reactions were incubated overnight (16 h) in the following concentrations: 3 µM HUH-tag, 30 µM nucleic acid substrate, 50 mM 4-(2-hydroxyethyl)-1-piperazineethanesulfonic acid (HEPES), pH 8.0, 50 mM NaCl, 1 mM dithiothreitol (DTT), 1 mM MnCl_2_ at 37°C.

Every member of this superfamily employs a catalytic tyrosine to cleave and then covalently link to the ssDNA 3′ cleavage product via a 5′ phosphotyrosine linkage. These nucleases have been co-opted for applications in biotechnology as functional fusion partners (HUH-tags) that enable facile and robust sequence-directed protein–ssDNA bioconjugation via a simple reaction on an unmodified and inexpensive ssDNA oligonucleotide harboring its specific sequence [20, 22] (Fig. 1A). Indeed, HUH-tags have been applied in CRISPR-based genome engineering as a means by which to covalently tether a repair template to Cas9 in order to enhance homology directed repair for gene editing and knock-in [23–25], and in receptor-specific cell targeting of adeno-associated viruses with capsid-fused HUH-tags linked to ssDNA-conjugated guiding antibodies to control viral tropism [26, 27]. Moreover, HUH-tag utilities extend beyond genome engineering into a diversity of applications, ranging from DNA-coordinated enzyme assembly [28] to high-throughput DNA tension gauge tether-based tension profiling [29]. HUH-tags are amenable to these applications due to their modest size and their ability to rapidly and robustly form nonlabile sequence-directed covalent linkages with unmodified ssDNA.

We have discovered that a minority of viral replication-initiating HUH-endonucleases from the *Circoviridae* family are able to form promiscuous sequence-directed covalent bonds to RNA, but with greatly reduced efficiency in comparison to cognate reactions with ssDNA substrates. Here, we describe the engineering and extensive *in vitro* characterization of a series of laboratory-evolved RNA-interacting HUH-tags derived from the HUH-endonuclease domain of the rep from Porcine Circovirus 2 (PCV2). Interestingly, the Ting lab published a preprint describing engineering the same HUH-endonuclease for RNA-linking activity soon after ours (now published [30]). These engineered enzymes provide a facile and efficient means by which to covalently link fusion proteins of interest to unmodified RNA substrates of interest in a sequence-specific manner, potentially enabling a host of new applications across a diversity of fields.

## Materials and methods

### Design and amplification of mutagenic libraries

Rep–RNA interaction engineering libraries were designed for site saturation mutagenesis of residues in proximity to ssDNA in co-crystal structures, starting with the ssDNA bridging motif (sDBM), which is known to impart sequence specificity in Reps. A maximum of six residues were mutagenized with ‘NNK’ codons (which include all 20 amino acid possibilities plus a single stop codon) in a single library, producing a theoretical library size of 21^6^, or roughly 85.8 million unique amino acid sequences. Alternative ambiguous codons, such as ‘DBK’, were also used to limit residue variation to a subset of amino acids defined from the results of previous engineering stages. Residues showing strong convergence to a single amino acid at a given position would typically be excluded from further rounds of mutagenesis.

Mutagenic libraries were produced in a similar fashion as described by Lambert *et al. *[31]. Briefly, insert libraries were designed with ∼100 base pair overlap with the 5′ and 3′ cleaved ends of the pETcon yeast surface display vector. Assembly polymerase chain reaction (PCR) was used to generate the mutagenic inserts, which produces a long double-stranded DNA (dsDNA) sequence from a collection of short overlapping top- and bottom-strand oligonucleotides. The collection of oligonucleotides used for the assembly reaction was designed by hand to generally consist of 60 bases and have annealing temperatures of 60°C to preceding and proceeding primers in the assembly. Site saturation mutagenesis at desired positions was achieved through the introduction of longer mutagenic oligonucleotide ultramers (IDT) in place of the shorter oligonucleotides spanning a region of interest.

Assembly inserts were produced through two sequential PCR amplifications with 2× CloneAmp HiFi PCR premix (Takara). Both reactions were performed in 25 μl volumes, with the first reaction consisting of 1 µl of a 1 µM mixture of the coding region-spanning assembly oligonucleotides and the 2× mastermix, and the second reaction consisting of 2.5 µl each of long forward- and reverse-primers—both with ∼100 base pairs of homology to the pETcon vector—and 2.5 µl of the first reaction (nonpurified) as a template for amplification. The second reaction was then separated by electrophoresis on a 1% agarose gel and purified via gel extraction. The purified mutagenic insert was then further amplified through numerous additional PCR amplifications using the same forward and reverse primers and purified via either PCR cleanup or gel extraction. Purified mutagenic insert products were pooled and stored at −20°C for future use.

### Media preparation

2× YPAD rich growth media; recipe for one liter: 20 g Bacto Yeast Extract (Thermo Fisher), 40 g Bacto Peptone (Fisher Scientific), 40 g D-glucose (Fisher Scientific), and 100 mg of Adenine hemisulfate (Sigma–Aldrich) dissolved in 990 ml of water, brought to a pH of 6.0 and autoclaved. Once cool, 10 ml of 100× pen/strep antibiotic solution (Thermo Fisher) and 500 μl of kanamycin at 50 mg/ml (Fisher Scientific) was added.

### Yeast transformation, culture, and induction of surface expression

The amplified mutagenic libraries were assembled into the pETcon plasmid using the yeast’s natural ability to perform homologous recombination by transformation with a mixture containing cleaved vectors and inserts with ∼100 base pairs of homology to its 5′ and 3′ ends. This results in a Gibson assembly-like recombination of the mutagenic inserts into the surface display expression plasmid via yeast recombination, consolidating plasmid library assembly and transformation into a single step. Mutagenic libraries were transformed into EBY100 *Saccharomyces cerevisiae* (ATCC) using a scaled-up version of the lithium acetate method. Briefly, the night prior to a transformation a small frozen aliquot of nontransformed yeast was cultured in 25 ml of nonselective growth media (2× YPAD). The next morning, this overnight culture was used to inoculate 100 ml of fresh 2× YPAD in a 500 ml baffled shake flask at 30°C shaking at 250 revolutions per minute (RPM) until the culture reached a density of around 100 million cells/ml. A total of 2.5 billion cells (∼25 ml) were then transferred to a 50 ml conical tube, spun down for 5 min at 3.5K G, and then resuspended in sterile water. Resuspended cultures were spun down a second time and resuspended in a transformation mixture composed of 2.4 ml 50% PEG 3, 350 (Sigma–Aldrich), 360 µl 1 M lithium acetate (Sigma–Aldrich), 500 μl of denatured salmon sperm DNA (Sigma–Aldrich), and a 340 μl mixture of ∼20 µg of the cleaved pETcon yeast surface display plasmid and ∼20 µg of mutagenic insert. Cells resuspended in the transformation mixture were then transferred to a 15 ml culture tube and incubated in a water bath at 42°C for 35 min, gently mixing every 5 min. Post heatshock, the culture was transferred to a 50 ml conical containing a 25 ml 50:50 mixture of selective media and 20% w/v glucose solution for recovery. Following this, the recovery mixture was added to 500 ml of selective media + glucose in a 2 l baffled shake flask and shaken at 250 RPM at 30°C overnight.

The following morning, yeast cells equal to roughly 50× the theoretical diversity of the library were washed in water and passaged into fresh selective media + raffinose and shaken at 250 RPM at 30°C for culture prior to induction. Once the culture reached a density of roughly 90–120 million cells per ml, the desired number of cells are again washed in water and transferred to selective media + galactose at a density of roughly 30 million cells/ml and left at room temperature overnight for induction of cell surface display.

### Protein engineering

Induced yeast cultures surface-expressing mutagenic libraries were sorted via two rounds of magnetic-activated cell sorting (MACS) with biotinylated substrates (Supplementary Tables S1 and S2) and magnetic streptavidin beads for functional enrichment, and a final round of fluorescence-activated cell sorting (FACS) with AF647-labeled substrates gated to sort out highly functional variants. The nucleic acid substrates used for selection were RNA versions of simplified *Cressdnaviricota* origins of replication (Ori) with 3 to 4 DNA bases appended to the 5′ and 3′ ends (IDT) in order to enhance the stability of an otherwise unmodified RNA substrate.

Briefly, for MACS selection, 1.5 billion induced yeast cells were washed two times with a washing buffer (500 mM KCl, 10 mM NaCl, 50 mM 4-(2-hydroxyethyl)-1-piperazineethanesulfonic acid [HEPES], pH 7.5) and then were resuspended at 100 million cells per ml in a reaction buffer (50 mM NaCl, 50 mM HEPES, pH 8.0, 1 mM MnCl_2_, and 100 nM of a 3′ biotinylated selection substrate) for subsequent incubation and reaction/covalent linkage for 30 min at 37°C. Post-incubation, cells were washed twice in washing buffer and resuspended at 100 million cells per ml in a bead-binding buffer [20 mM Tris, pH 7.5, 500 mM NaCl, 1 mM ethylenediaminetetraacetic acid (EDTA)] and 1 mg of hydrophilic streptavidin magnetic beads (NEB) equilibrated into the same buffer. Yeast-bead solutions were rocked at 4°C for 2 h before selection with a magnetic rack by pulling down the magnetic beads and removing the nonbound yeast culture supernatant. Magnetic beads were washed twice with the bead-binding buffer and resuspended in 5 ml of selective media with glucose for subsequent growth for 16 h. Following this, the cultures were spun down and resuspended with 1 ml of selective media with raffinose, incubated on a magnetic rack at room temperature for 5 min, and the nonbound supernatant was transferred to fresh selective media with raffinose for further growth and induction.

Briefly, for FACS selection, the desired number of induced yeast cells (typically ∼100 million) were washed two times with the washing buffer and were then resuspended at 100 million/ml in a reaction buffer (50 mM NaCl, 50 mM HEPES, pH 8.0, 500 μM MnCl_2_, and 50 nM AF647-labeled substrate) for subsequent incubation and reaction at 37°C for 30 min. Post-incubation, cells were washed twice in washing buffer and resuspended at 100 million cells/ml in a staining buffer [200 mM KCl, 10 mM NaCl, 50 mM HEPES, pH 7.5, 1:100 dilution of a fluorescein isothiocyanate (FITC)-labeled anti-Myc antibody (Immunology Consultants Laboratory, ICL)] and then rocked in the cold room for up to 2 h. Following this, cells were spun down and resuspended in this same staining buffer at roughly 50 million cells/ml and held on ice until sorted on a BD FACSAria II P0287 cell sorter at the University of Minnesota flow cytometry resource. Cells with the highest induction (FITC) and activity signals (AF647) were targeted for sorting. Flow sorted cells were then transferred to fresh selective media + glucose and allowed to shake overnight at 250 RPM at 30°C prior to further culture and manipulation.

### Flow cytometry analysis of expression and activity on the yeast surface

Enzymes displayed on the yeast surface were analyzed for activity and expression in a manner similar to that of FACS selection, but on a much smaller scale. Stained cells were analyzed on a BD Accuri benchtop flow cytometer (BD) using the FITC and AF647 channels with no compensation necessary.

### Next-generation sequencing and analysis of enzyme variants

Following flow sorting, cells were further cultured for subsequent analysis and plasmid extraction (Zymo). The enzyme coding sequences were amplified from the plasmids using PCR with primers containing sequencing adapters. The amplicons were then purified via gel extraction and sent for next-generation sequencing (NGS; Amplicon-EZ, Azenta).

### Molecular cloning

The wild-type (WT) 6xHis-SUMO-Rep PCV2 plasmid was generated in a previous study [20]. Briefly, a WT Rep sequence was cloned into the pTD68/6xHis-SUMO expression vector as a geneblock (IDT) with the In-Fusion HD Cloning Kit (Takara) using BamHI and XhoI restriction sites (NEB). Engineered variant plasmids (Supplementary Tables S1 and S2) for protein expression were either generated in an identical manner or through site-directed mutagenesis using the same cloning kit per manufacturer instructions. Enzymes containing point mutations were also created via site-directed mutagenesis as described above. Each plasmid was sequence confirmed with Sanger sequencing (Genewiz) prior to use.

### Protein expression and purification

Rep constructs, including the engineered variants, were expressed in BL21(DE3) competent *Escherichia coli* cells (Agilent) in a 1 l volume with Luria-Bertani (LB) broth. Temperature was reduced from 37°C to 18°C after the culture OD600 reached 0.8 and cells were induced with 0.5 mM isopropyl β-d-1-thiogalactopyranoside (IPTG) (Sigma–Aldrich) and incubated overnight. Cells were lysed in a lysis buffer (250 mM NaCl, 50 mM HEPES, pH 8.0, 1 mM EDTA) with a complete protease inhibitor tablet (Pierce) and pulse sonicated at 4°C. Clarified supernatant was batch bound with Ni-NTA HisPur agarose beads (Thermo Fisher) for 1 h, loaded onto a gravity column, washed with 30 column volumes of wash buffer (250 mM NaCl, 50 mM HEPES, pH 8.0, 1 mM EDTA, 30 mM imidazole), and finally eluted with elution buffer (250 mM NaCl, 50 mM HEPES, pH 8.0, 1 mM EDTA, 300 mM imidazole). Purification was assessed via SDS–PAGE gel analysis and protein-containing fractions were dialyzed (250 mM NaCl, 50 mM HEPES, pH 8.0, 1 mM EDTA, 1 mM dithiothreitol [DTT]) overnight at 4°C. Protein was further purified and buffer exchanged using the Superdex 300 Increase 10/300 GL (GE Healthcare) size exclusion column into the lysis buffer for storage and characterization. For the production of SUMO-free protein, ∼30 µg 6xHis-ULP1 (SUMO-specific protease) per liter of culture was added to the protein samples prior to overnight dialysis. Following this, protein samples were then batch-bound a second time with Ni-NTA HisPur agarose beads to remove cleaved 6xHis-SUMO and 6xHis-ULP1 for subsequent buffer exchange as described above. rHUH was cloned into the same pTD68/6xHis-SUMO expression vector and purified using the same strategy as the our engineered variants E1 and E2.

### In vitro HUH cleavage reactions

*In vitro* oligonucleotide cleavage reactions were carried out using final concentrations of 3 µM N-terminal SUMO-tagged Rep variant and 30 µM single-stranded nucleic acid Ori substrate (IDT) in a cleavage buffer of 50 mM NaCl, 50 mM HEPES, pH 8.0, 1 mM DTT, and 1 mM MnCl_2_ and incubated overnight. Alternatively, restrictive reactions were carried out using final concentrations of 3 µM N-terminal SUMO-tagged Rep variant and 15 µM single-stranded nucleic acid Ori substrate in a cleavage buffer of 50 mM NaCl, 50 mM HEPES, pH 8.0, 1 mM DTT, and 50/1000 µM MnCl_2_ and incubated for 30 min unless otherwise stated. Reactions were incubated at 37°C and quenched with 10 mM EDTA before subsequent denaturation and SDS–PAGE analysis. Background RNA was purified from cultured Drosophila DL1/S1 cells, and total RNA was isolated using standard kits.

### Fluorescence polarization assays

The affinity of engineered and WT Reps for ssDNA and ssRNA were measured via fluorescence polarization as previously described [32]. 3′ fluorescein (FAM) labeled nucleic acid substrates (IDT) were used in all experiments (Supplementary Tables S1 and S2). N-terminal SUMO-tagged catalytically inactive Reps were titrated via 1:2 serial dilution starting at 1 µM into a 10 nM solution of a FAM-labeled substrate in a binding buffer of 50 mM NaCl, 50 mM HEPES, pH 8.0, 0.05% Tween-20, 1 mM DTT, and 5 µM MnCl_2_. A single binding reaction was assembled in quadruplicate using four rows across a 96-well plate and three independent replicates of this reaction were performed across separate days. Reactions were incubated for 30 min at room temperature to ensure equilibrium and centrifuged at 2000 G for 2 min before measurement. Mean dissociation constants (*K*_D_) were calculated across three separate reactions (*n* = 12) using the equations described below. In order to calculate polarization from parallel and perpendicular fluorescence intensity values, data were fit to the following equation:

Polarization = (Ipar − Iper)(Ipar + 2Iper)

Where Ipar equals parallel fluorescence intensity and Iper equals perpendicular fluorescence intensity. In order to calculate *K*_D_ under nonsaturating ligand conditions, data is fit to a quadratic model:

A = (Amax − Amin)(2 [Dt]([Pt]+ [Dt] + *K*_D_) − √([Pt] + [Dt] + *K*_D_)2 − 4 [Pt] [Dt])+ Aminwhere [Dt] is the total amount of labeled ssDNA, [Pt] is the total amount of protein, kD is the dissociation constant, Amax is the anisotropy ceiling, and Amin is the anisotropy floor. All anisotropy data were collected using a Synergy Neo2 Hybrid Multi-Mode Plate Reader and the Green FP Filter Cube (Agilent) with an excitation wavelength of 485 nm (20 nm bandwidth). Emissions of 528 nm were measured at ambient temperature. Measurements were fit and plotted in GraphPad Prism (v. 6.07) according to the above equations.

### Molecular beacon cleavage assays

The cleavage rate of engineered and WT Rep variants was evaluated with a molecular beacon assay using either a ssDNA or ssRNA probe harboring an Ori-derived sequence labeled with a 5′ IowaBlack-FQ quencher and a 3′ FAM (Q/F, IDT). All cleavage reactions were performed in a cleavage buffer of either 50 mM NaCl (for RNA cleavage) or 500 mM NaCl (for DNA cleavage), 50 mM HEPES, pH 8.0, 0.05% Tween-20, 1 mM DTT, and 50 µM MnCl_2_. N-terminal SUMO-tagged Rep variants were diluted with the cleavage buffer to 2 µM and the Q/F substrate was diluted to 200 nM in an identical buffer. In a black 96-well plate, 100 µl of the Q/F substrate was added to each well and inserted into a Synergy Neo2 Hybrid Multi-Mode Plate Reader (Agilent). Using the injector module, 100 µl of a Rep variant was added to each well, initiating the reaction. Fluorescent signal was collected by the plate reader using the monochromator function with an excitation wavelength of 485 ± 20 nm and emission collected using a 528 ± 20 nm bandpass filter at room temperature. Emission values for all eight reactions were collected every 5 s for 15 min. At a minimum, three independent replicates were collected for each condition.

### Differential scanning fluorimetry

Differential scanning fluorimetry (DSF) experiments were conducted using a CFX96 Touch™ Real-Time PCR Detection System (Bio-Rad) with FRET channel detection. The assay was performed in skirted qPCR plates with a final reaction volume of 35 μl per well. The reaction mixture consisted of 2 μM enzyme, 600 mM NaCl, 20 mM HEPES (pH 8.0), and 10× SYPRO Orange dye (Thermo Fisher). A master mix was prepared for each protein of interest, containing 2× DSF buffer (1.2 M NaCl, 40 mM HEPES, pH 8.0), 30 μM protein stock, and water. SYPRO Orange dye was diluted separately to a 70× working solution using 2× DSF buffer and water. The plate was prepared with five replicates each of water-only, buffer-only, and protein samples. Four out of five wells for each condition received 5 μl of 70× SYPRO Orange dye, with one well left dye-free as a control. The plate was then centrifuged at 2000 G for 2 min. Thermal denaturation was measured using a temperature gradient from 20°C to 95°C, with a ramp rate of 1°C/min. Melting temperatures (Tm) were determined from the first derivative of the thermal denaturation profiles using GraphPad PRISM (v. 6.07).

### Phylogenetic tree construction and visualization

The phylogenetic tree depicted in Fig. 1A was generated by first downloading all of the complete rep protein sequences available across each of the seven families in *Cressdnaviricota* (*Bacilladnaviridae, Circoviridae, Geminiviridae, Genomoviridae, Nanoviridae, Redondoviridae*, and *Smacoviridae*) from NCBI Virus, clustering them at 90% using MMseqs2 [33] to remove highly similar or identical sequences, MAFFT [34] aligning the remaining protein sequences, constructing a phylogenetic tree using FastTree2 [35], and visualizing the tree in Interactive Tree of Life.

### Amino acid enrichment sequence logo plotting

**Figure 2.**
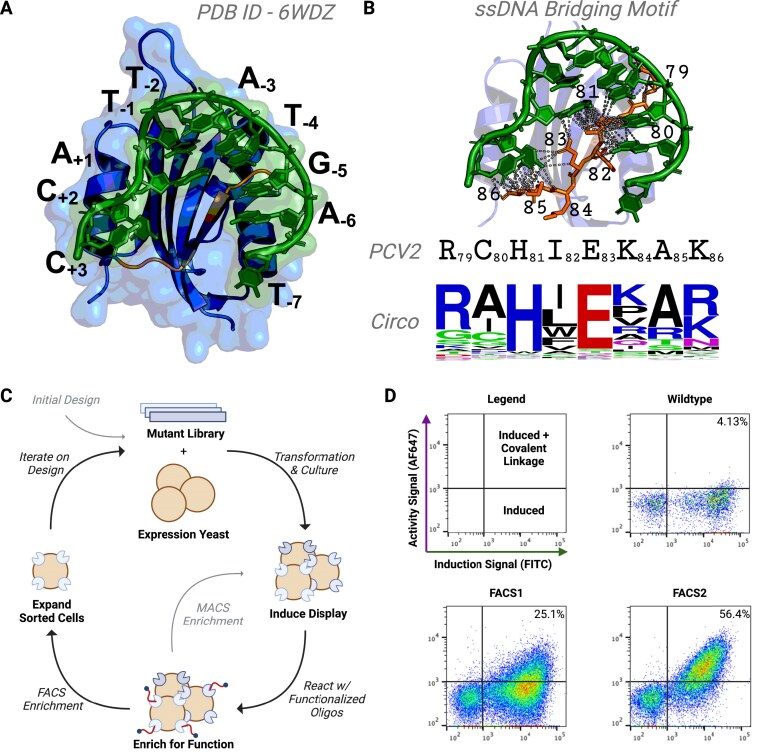
Overview of Directed Evolution. (**A**) Depiction of the co-crystal structure of the rep enzyme PCV2 (blue) in complex with an ssDNA mimic of its cognate origin of replication (green). The specific bases across each position of the bound ssDNA 10-mer are noted in black and the beta strand comprising the sDBM is recolored in orange. (**B**) Close up view of the sDBM in PCV2 and the extensive contacts it makes with its cognate ssDNA substrate. The motif’s residue identities and positions are indicated directly underneath the structure in black along with a sequence logo representing the natural variation across the positions in this motif across *Circoviridae*. (**C**) High-level graphical overview of the yeast surface display-based directed evolution strategy employed to engineer PCV2 towards activity on noncognate RNA substrates. (**D**) Results summary of yeast surface display directed evolution. The flow cytometry plots indicate the population of yeast inducing enzyme expression (shifts on the X axis) and covalently linking to fluorescently labeled RNA (shifts on the Y axis) for the WT enzyme as well as the heterogeneous results of the FACS sorting from the first and second rounds of engineering (FACS1 and FACS2).

**Figure 3.**
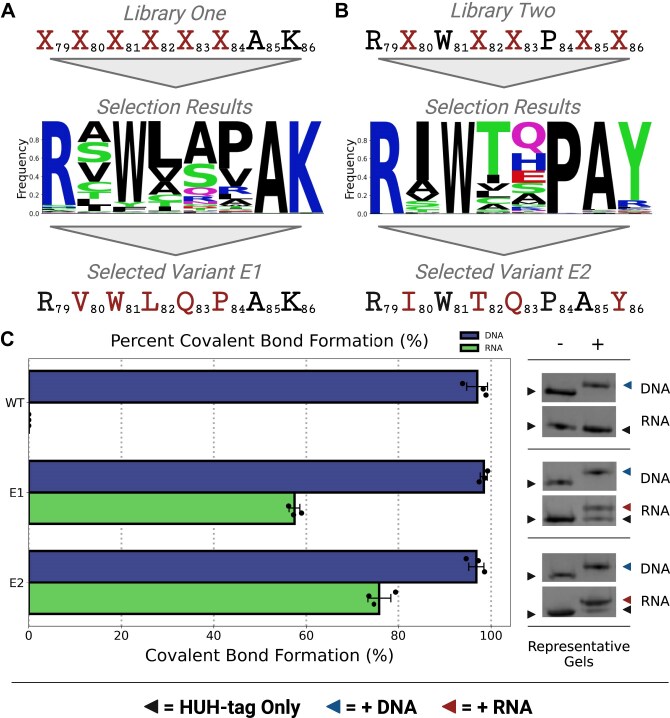
Improved protein–RNA covalent linkage in engineered reps. (**A, B**) High-level overview of mutagenic library design and selection in the first round of sDBM engineering. The ‘X’ positions under ‘Library’ indicate the residue positions replaced with degenerate codons, the ‘Selection Results’ shows a sequence logo that indicates enrichment following FACS sorting of the library, and the ‘Selected Variant’ shows the mutant sDBM selected following selection and analysis. (**C**) The *in vitro* HUH-tag bioconjugation reactions visualized via SDS–PAGE in triplicate represented as bar plots for both DNA (top) and RNA (bottom) substrates with the WT, E1, and E2 enzyme variants next to representative SDS–PAGE gels that indicate covalent linkage as an increase in molecular weight. Error bars represent the standard deviation of replicates. Reactions were performed in final concentrations of 3 μM HUH-tag and 15 μM nucleic acid substrate in 50 mM HEPES, pH 8.0, 50 mM NaCl, 1 mM DTT, and 50 μM MnCl_2_ for 30 min at 37°C.

The residue enrichment sequence logos shown in Figs 2B and 3A and B were generated with custom python scripts using the Logomaker [36] package. Briefly, for Fig. 2B, we downloaded all available rep sequences from *Circoviridae* from NCBI Virus clustered them at 90% using MMseqs2 [33] to remove highly similar or identical sequences, MAFFT [34] aligned them, and then trimmed the alignments using trimAI [37] to remove any columns consisting of gap characters in 90% or more of sequences. Following this, we plotted the region of the alignment corresponding to the sDBM as an enrichment logo. Further, in Fig. 3A and B, we analyzed NGS data from selections using the Biopython [38] package and a custom python script that quantifies the enrichment of each unique mutant variant present in the population. Following this, we plotted the region of the alignment corresponding to the sDBM as an enrichment logo.

### HUH-seq ssDNA library cleavage, library preparation, and sequencing

A 97-nt ssDNA library with a central seven-base randomized region flanked by primer binding sites at both its 5′ and 3′ ends was synthesized by IDT. Positions falling outside of the randomized region and the four bases immediately upstream of it contained phosphorothioate to limit off-target cleavage in a nonlibrary location. Cleavage of the library was carried out in triplicate with 3 μM Rep and 300 nM ssDNA library in 50 mM HEPES, pH 8.0, 50 mM NaCl and either 1 mM MnCl_2_ or 50 µM MnCl_2_ for 1 h at 37°C. Following this, the enzymes were immediately heat-inactivated by boiling at 95°C for 5 min. The reactions were then diluted 10-fold in water and amplified by primers containing unique barcodes to identify specific reactions in NGS data. Following this, barcoded amplifications were gel purified and again amplified with primers containing sequencing adapters. The resulting product was a 245 bp dsDNA amplicon run on a 1% agarose gel and purified via gel extraction. Finally, barcoded samples were pooled and then sequenced a single Illumina HiSeq lane (350 000 000 paired-end reads, Azenta) spiked with 30% PhiX.

### HUH-seq read count reduction analysis and sequence specificity logo plotting

Raw NGS reads were processed using the Biopython package [38]. The reads were first de-multiplexed into their own FASTQ files using their associated barcode sequences. Following this, the randomized portions of the library were extracted (k-mers) from each of the files. Mean frequencies for each of the 16 384 k-mers from the randomized 7-mer library were generated for the reference library and each of the enzyme treatment libraries across their three replicates excluding the low Mn^2+^ condition of WT PCV2 where replicate one correlated poorly with replicates two and three (Supplementary Fig. S3). Each treatment was compared against the reference to calculate a mean percent reduction (reference—treatment/ reference) for each of the k-mers. This percent reduction data was used to produce sequence logos indicative of the sequence specificity of the assayed enzyme in each of the treatment libraries. The quality and consistency of each of the libraries was assessed by evaluating the correlation of read counts per k-mer across each of the replicates (Supplementary Fig. S3).

## Results

### Identifying promiscuous protein–RNA covalent linkage

In our previous work, we introduced HUH-tags as functional fusion partners capable of mediating sequence-directed protein–ssDNA covalent bioconjugation in a single step [20, 22]. Recently, the reps have emerged as the preferred HUH-tag type due to their modest size, compact cleavage motifs, and robust reactivity [22]. Moreover, our extensive knowledge of their sequence specificity, coupled with the recent development of computational tools to aid in their substrate design [39], have further simplified their deployment and increased the utility of rep-based HUH-tags. While reps can be identified in a wide diversity of bacterial, archaeal, and viral species, HUH-tags are primarily co-options of reps belonging to the ssDNA virus phylum *Cressdnaviricota* [40] (Fig. 1A)—typically from the *Circoviridae, Geminiviridae*, and *Nanoviridae* families. Interestingly, we recently discovered that reps originating from *Circoviridae* exhibit some promiscuity which enables their interaction with ssRNA, albeit with dramatically lower efficiency in comparison to reactions with cognate ssDNA substrates (Fig. 1B and Supplementary Fig. S1).

HUH-tags typically form covalent adducts with cognate DNA sequences in minutes at 37°C in the presence of divalent cations such as Mn^2+^. To enhance our chances of observing promiscuous reactions of HUH-tags with RNA, we performed overnight *in vitro* reactions with a panel of HUH-tags—three derived from the circoviruses PCV2, duck circovirus and columbid circovirus, and one from the geminivirus wheat dwarf virus (WDV). SDS–PAGE analysis showed that overnight reaction with DNA oligonucleotide substrates harboring the enzymes’ specific cleavage motif yielded the expected robust protein–ssDNA bioconjugate formation across all four enzymes tested (Fig. 1B and Supplementary Fig. S1). Intriguingly, reps derived from *Circoviridae*, but not *Geminiviridae*, were also capable of forming sequence-directed covalent linkages with RNA, but with dramatically lower efficiency in comparison to reactions with cognate ssDNA substrates (Fig. 1B). Importantly, these reactions were performed in highly permissive conditions to maximize our ability to identify promiscuous activity.

The discovery of promiscuous activity on RNA substrates in *Circoviridae*-derived reps presents an exciting opportunity to develop RNA-interacting HUH-tags—fusion proteins capable of mediating simple and efficient sequence-directed protein–RNA covalent linkage to unmodified RNA substrates. We have previously demonstrated that reps from PCV2 and WDV are amenable to rational engineering [20], making PCV2 the ideal enzyme scaffold to enhance the HUH–RNA interaction through directed evolution using yeast surface display. While enzymes are typically engineered rationally through site-directed mutagenesis and *in vitro* screening [41], high-throughput methods, such as yeast surface display [42] or droplet microfluidics [43], have also been employed successfully across a wide variety of examples. Indeed, yeast surface display has even been used to engineer other bioconjugation-mediating enzymes, such as sortase [44], a transpeptidase that catalyzes sequence-specific intermolecular protein ligation. Moreover, this method is commonly used to engineer meganucleases for targeted gene editing applications [45], further highlighting the versatility and effectiveness of yeast display in the directed evolution of a diverse range of enzymes. The self-labeling nature of reps makes them ideal candidates for yeast display methods, which often rely on fluorescence-based selection using flow cytometry, as engineered variants with enhanced properties can accumulate higher fluorescence in a gain-of-signal (i.e. ‘signal on’) selection scheme.

### Developing a directed evolution approach for rep engineering

Our previous work identified a critical motif involved in mediating ssDNA substrate interaction across the HUH-endonuclease superfamily, the sDBM [20]. This motif has been shown to be a major determinant of sequence specificity in reps, positioning it as an ideal candidate for mutagenesis. Indeed, a recently elucidated co-crystal structure of PCV2 in complex with its cognate substrate highlights the importance of this simple beta sheet structure in ssDNA positioning and stabilization, revealing that this motif makes extensive contacts across its bound substrate (Fig. 2A and B). Importantly, there is considerable natural variability across this region, even within the *Circoviridae* family (Fig. 2B). We reasoned that mutating this motif could reposition or remodel the active site to better accommodate noncognate RNA substrates in PCV2 and further enhance the efficiency of protein–RNA covalent linkage of this enzyme. In order to extensively sample mutagenized sDBM motifs that could enhance RNA interaction, we developed a yeast surface display-based platform to perform selections in high throughput. Yeast surface display is a powerful tool for directed evolution that employs *S. cerevisiae* to establish a link between genotype (enzyme coding sequence) and phenotype (enzyme function). In this system, mutant enzymes are tethered to the yeast cell surface through plasmid-driven expression as fusions to a cell wall-anchoring protein which facilitates their export and attachment to the cell’s exterior. Because reps are self-labeling enzymes, high-performing enzymes are expected to accumulate increased functionalized substrate (either biotinylated or fluorescently labeled RNA) on the cell surface. Consequently, cells displaying superior enzymes can be effectively selected and enriched through MACS with streptavidin beads or FACS (Fig. 2C).

In initial tests, we induced expression of the WT PCV2 enzyme on the surface of yeast and reacted it for 30 min with fluorescently labeled RNA substrates in the presence of saturating Mn^2+^ to assess if baseline promiscuous activity is detectable in this context. Flow cytometry analysis revealed that ∼4% of the total yeast population displayed proteins labeled with fluorescent RNA (Fig. 2C and D). However, the magnitude of the fluorescent signal was quite low, indicating that the reaction efficiency of the WT enzyme on RNA was modest, but detectable, which is consistent with our previous *in vitro* observations (Fig. 1B).

While the WT enzyme’s RNA-linking activity was relatively poor on the surface of yeast, it provided a detectable signal that served as a baseline for selecting variants with higher levels of activity. Thus, we designed site-saturation mutagenesis libraries to screen for enzyme variants with enhanced function on RNA substrates using our yeast surface display system. Specifically, we synthesized mutagenic libraries by using assembly PCR [46] to replace residues in the sDBM with degenerate codons that encode all twenty amino acids and one stop codon. This strategy enables comprehensive screening of all potential combinations of mutations across positions of interest, facilitating an exhaustive search of the local sequence space to identify enzymes with optimal function. In our initial library, we comprehensively mutated a stretch of six residues (positions 79–84) on the sDBM (Fig. 3A and Supplementary Fig. S2). We amplified and assembled the mutagenic library into yeast by leveraging the organism’s natural ability to mediate plasmid recombination [47]. To select for enzymes with optimized function, we performed two initial rounds of MACS with increasing stringency to enrich the population for highly functional variants followed by a single round of FACS, specifically isolating the cells with the highest fluorescent signals for both induction and activity. Post-selection analysis of the heterogeneous population via flow cytometry revealed that the proportion of cells with detectable signal had increased from 4% to 25% (Fig. 2C and D). Subsequent NGS of the plasmids from the sorted population identified a high degree of convergence towards a specific mutation, H81W, which likely serves as the key driver of improved function in this initial round of engineering (Fig. 3A). We selected an initial improved variant (E1) based roughly on consensus from a sequence logo indicating the enhanced population’s residue enrichment for each of the six mutagenized positions in the sDBM (Fig. 3A). Building upon the success of our initial round of engineering, we proceeded with a second iteration of mutagenesis, further screening both positions that did not converge to a single residue and positions further downstream in the sDBM. Specifically, we selected positions 80, 82, 83, 85, and 86 for further mutagenesis while stabilizing positions that demonstrated high convergence in the previous round. Specifically, we fixed residues 79 to arginine, 81 to tryptophan, and 84 to proline (Fig. 3B). We performed MACS and FACS enrichment and selection as described in the preceding round to isolate variants with further enhanced function. Strikingly, post-sort characterization of the heterogeneous population via flow cytometry revealed a substantial increase in the proportion of cells with detectable signal, jumping from roughly 25%–56% (Fig. 2C and D). Subsequent NGS and analysis once again demonstrated strong convergence at a specific position that may be responsible for the enhanced RNA interaction in the sorted population. In particular, a K86Y mutation exhibited by far the strongest convergence to a non-WT residue and is likely the primary driver of improved activity in this second round of engineering (Fig. 3B). We again selected a further improved variant (E2) based on consensus from the NGS analysis (Fig. 3B).

### Directed evolution yields engineered variants with enhanced properties

Having successfully enhanced the activity of our enzyme on the surface of yeast by tenfold, we sought to characterize performance *in vitro* to determine if our selected engineered variants are suitable for application as RNA-interacting HUH-tags. We recombinantly expressed and purified the selected E1 and E2 enzymes to test their ability to covalently link to both DNA and RNA substrates *in vitro*. To better differentiate the function of WT and engineered variants, we implemented dramatically more stringent reaction conditions. Specifically, we reduced the substrate concentration from 30 µM (10× substrate:enzyme ratio) to 15 µM (5× ratio). Additionally, we decreased the MnCl_2_ concentration from 1 mM to 50 µM. Finally, we shortened the reaction time from overnight to 30 min. Remarkably, while ssDNA covalent linkage remained roughly consistent across the WT, E1, and E2 enzymes, activity on the noncognate RNA substrate enhanced dramatically, improving from undetectable levels in the WT enzyme under restricted conditions to roughly 60% and 80% in E1 and E2, respectively (Fig. 3C).

### Binding and cleavage kinetics of engineered proteins

**Figure 4.**
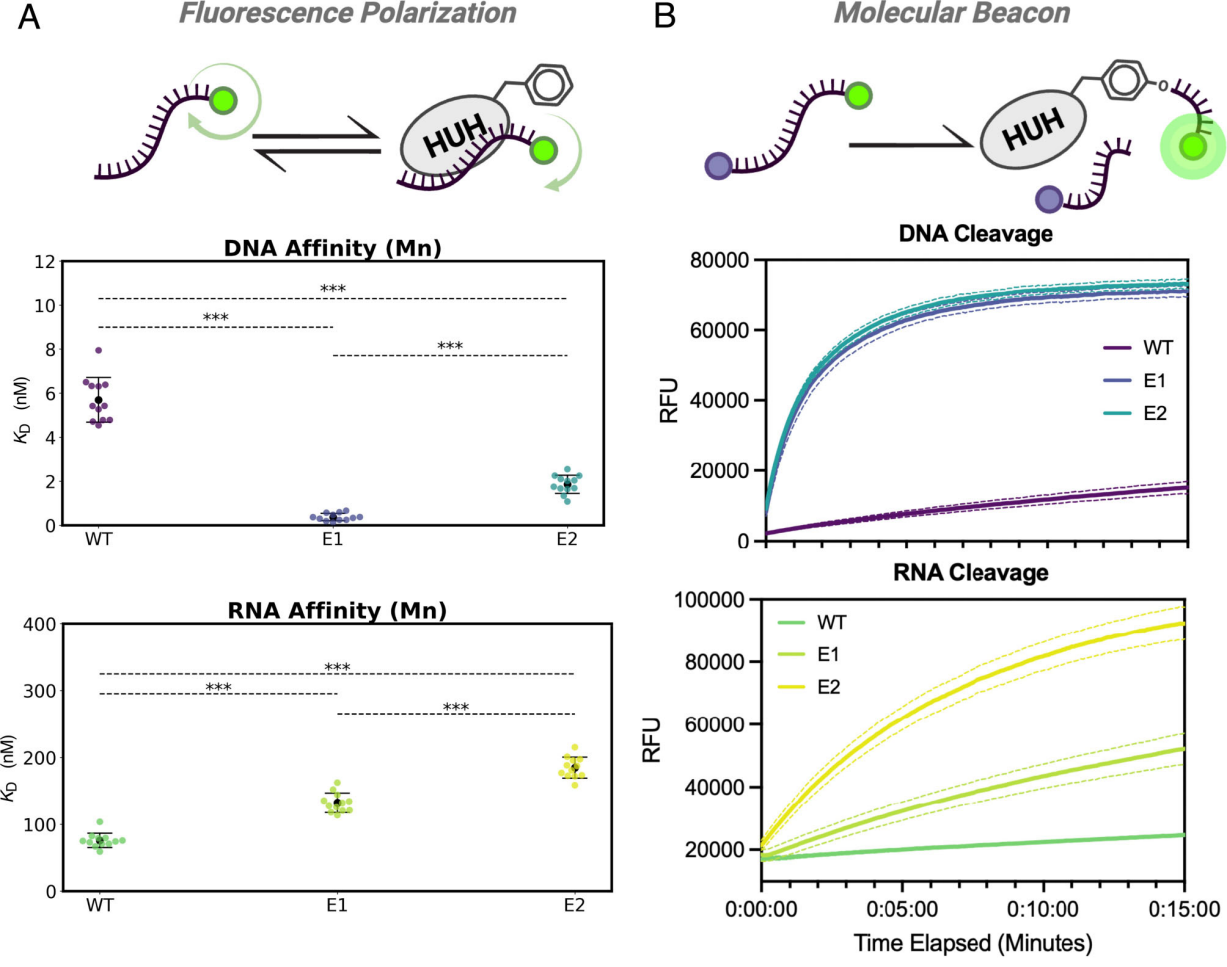
Binding affinities and cleavage kinetics of WT and engineered enzymes across nucleic acid substrates. (**A**) Graphical overview of the fluorescence polarization assay to gauge binding affinity, and the results of this assay with the WT, E1, and E2 variants on DNA (purple/blue color scheme) and RNA (green/yellow color scheme) substrates. Data are presented as mean of individual experiments (black dots) ± standard deviation. Statistical significance was determined using one-way analysis of variance (ANOVA) with Tukey’s multiple comparisons test (GraphPad Prism). *n* = 12 biological replicates. ***P <.0001. (**B**) Graphical overview of the molecular beacon cleavage assay to gauge cleavage kinetics, and the results of this assay with the WT, E1, and E2 variants on DNA (purple/blue color scheme) and RNA (green/yellow color scheme) substrates. Each solid curve represents the average values across three runs and the dashed curves represent their variability.

We next examined the binding and cleavage kinetics of the engineered mutants toward their nucleic acid substrates. We first used a previously described fluorescence polarization-based assay [32] to quantify changes in affinity across our WT and engineered enzymes for both cognate ssDNA and noncognate ssRNA substrates. Interestingly, although the directed evolution efforts were aimed at enhancing catalysis on RNA, we observed a modest improvement in DNA binding affinity throughout the engineering process. The dissociation constant (*K*_D_) decreased from ∼6 nM in the WT enzyme to just under 1 nM in the E1 variant, ultimately stabilizing at nearly 2 nM in the E2 variant in the presence of Mn²⁺ as observed by fluorescence polarization (Fig. 4A and Supplementary Fig. S3). Counterintuitively, the affinity for RNA decreased modestly as the engineering progressed, with the *K*_D_ increasing from ∼90 nM in the WT enzyme to roughly 150 nM in the E1 variant, and finally reaching just under 200 nM in the E2 variant in the presence of Mn²⁺ (Fig. 4A and Supplementary Fig. S3C). Furthermore, evaluation of binding in the absence of the catalytically-required Mn^2+^ ion showed consistent trends (Supplementary Fig. S3A and B). The absence of metal had a large impact on DNA binding, reducing affinities roughly tenfold to roughly 30, 10, and 15 nM for WT, E1, and E2 enzyme variants, respectively. Interestingly, this condition had comparatively less effect on RNA binding, modestly reducing affinities to roughly 100, 150, and 300 nM for WT, E1, and E2, respectively, suggesting that RNA binding is less dependent on divalent ions.

We next sought to quantify the catalytic performance of the WT and engineered enzymes by comparing their nucleic acid cleavage kinetics. To do so, we adapted a previously described continuous molecular beacon assay [22, 32]. This assay uses single-stranded oligonucleotide substrates containing the enzyme’s cognate cleavage motif and flanked by a FAM fluorophore and Iowa Black quencher at the 3′ and 5′ ends, respectively. Cleavage separates the fluorophore from the quencher, producing a fluorescence increase that can be continuously monitored in a plate reader format.

Because the DNA substrate binds with very high affinity (low *K*_D_), reactions were performed at substrate concentrations above the binding constant, allowing the observed rate to approximate the catalytic rate constant (*k*_cat_). The optimal beacon concentration (∼100 nM) provided strong signal-to-noise while minimizing reagent consumption. In contrast, the RNA substrate binds with much lower affinity, precluding use of substrate concentrations high enough to saturate binding; therefore, this assay reports relative rather than absolute cleavage rates for RNA.

Prior to kinetic comparison, assay conditions were optimized to keep reactions within the dynamic range of the detector. The engineered variants exhibited such high catalytic efficiency on DNA substrates that reactions had to be deliberately slowed by addition of 500 mM NaCl, which reduces electrostatic interactions between the enzyme and nucleic acid, thereby weakening binding and lowering apparent rates. Metal titration experiments revealed striking effects on RNA cleavage: as shown in Supplementary Fig. S4, the E2 variant showed no detectable activity with Mg²⁺ but robust cleavage with Mn²⁺, with an apparent Mn²⁺ dissociation constant of ∼90 µM.

Under optimized conditions, we compared cleavage kinetics of WT and engineered enzymes using both DNA and RNA molecular beacons. The engineered variants cleaved DNA substrates dramatically faster than WT (Fig. 4B), a difference not apparent in single–time-point covalent linkage assays. Reactivity toward RNA also improved substantially across successive engineering rounds, increasing from nearly undetectable levels in WT to robust activity in later variants—consistent with endpoint linkage results. Because substrate concentrations were below the *K*_D_ for RNA, the measured rates likely underestimate the true catalytic step, as discussed above.

### Structure comparison and *in vitro* characterization confirms key mutations

We were surprised by the substantial shift in kinetics observed with cognate ssDNA substrates considering that this is not what we were intentionally selecting for. Intrigued by these unexpected results, we sought to better understand the consequences of the changes that we made to these enzymes throughout the engineering process.

By design, all mutations made throughout the engineering process were clustered in the sDBM, with E1 and E2 differing from the WT enzyme by five and six mutations, respectively (Supplementary Fig. S5). Mutagenic libraries were designed to modify key positions that make direct and indirect contacts with the ssDNA substrate in an attempt to remodel the active site to enable RNA interaction. In examination of the co-crystal structure of WT PCV2 in complex with its cognate ssDNA substrate [20], the effects of the key mutations revealed by sequencing can be considered in their local structural contexts. The H81W mutation likely improves the contact made between the enzyme and the +1 A, whereas the role of the K86Y mutation is seemingly less straightforward to interpret (Supplementary Fig. S5). Interestingly, the engineered variants are strongly enriched for specifically tyrosine at position 86, never phenylalanine, indicating the importance of the hydroxyl group, possibly in mediating a hydrogen bond in an unanticipated location (Fig. 3B and Supplementary Fig. S5). To confirm the importance of these two mutations, we reverted them back to their WT residues in context of the E2 enzyme and performed *in vitro* reactions on DNA and RNA substrates. The reversion of these two mutants had catastrophic effects on enzyme activity, highlighting their crucial roles in the enhanced performance of the engineered variants. Specifically, the W81H mutation in E2 nearly ablated RNA interaction altogether under restricted reaction conditions, whereas the Y86K mutation only modestly decreased activity compared to E2 (∼80% linkage to ∼75% in the point mutant). The combination of these two mutations brought RNA interaction to a nearly undetectable level, mirroring the behavior of the WT enzyme (Supplementary Fig. S5B). This likely indicates that the majority of our improved function resulted from these two mutations alone. Furthermore, another unanticipated consequence of the engineering process was the loss of thermal stability. Using differential scanning fluorimetry on WT and engineered versions of our enzymes, we found that the melting temperature (Tm) decreased from roughly 53°C in the WT enzyme to ∼50°C in E1 and further down to just under 45°C in E2, suggesting that our installed mutations may be destabilizing the enzyme (Supplementary Fig. S5C). Loss of stability is not uncommon in protein engineering endeavors and is frequently observed in meganucleases engineered using yeast surface display based directed evolution [48]. However, this finding further underscores the complex interplay between the introduced mutations and the overall protein structure and function and how even modest-seeming mutations can have unanticipated results.

### Extensive sequence specificity profiling with HUH-seq

**Figure 5.**
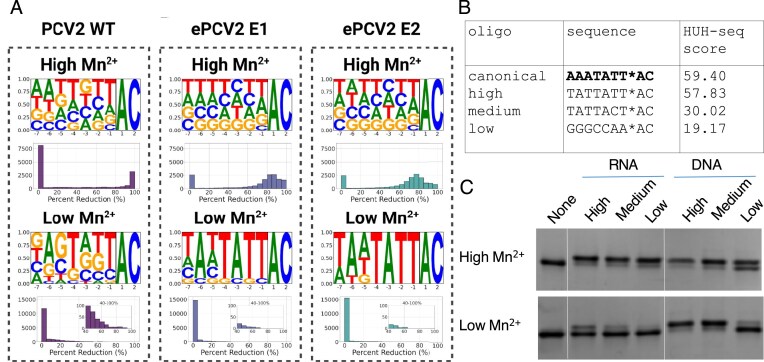
Extensive sequence specificity profiling and validation of engineered HUH-tags. (**A**) HUH-seq results measure cleavage of a partially random DNA oligo 5′ N_-7_N_-6_N_-5_N_-4_N_-3_N_-2_N_-1_*A_+1_C_+2_ 3′ by the WT and engineered PCV2 E1 and E2 variants in high (top) and low (bottom) Mn^2+^ conditions. Results are reported as weighted sequence logos indicative of sequence specificity and histograms representing the number of unique sequences cleaved at a given efficiency (i.e. percent reduction value) out of the 16 384 unique k-mers across conditions. (**B**) Oligo sequences with predicted high, medium, and low efficiency cleavage based on the HUHseq data at low Mn^2+^ concentrations. The HUH-seq score is the percent reduction of kmers compared to uncleaved controls. (**C**) DNA and RNA oligonucleotides with corresponding sequences were purchased and reacted with E2 at high and low Mn^2+^ conditions.

To determine whether enhanced RNA reactivity arose from improved overall catalytic activity or from altered sequence specificity, we profiled the engineered enzymes using our previously described HUH-seq assay [20] (Supplementary Fig. S6). HUH-seq is an NGS-based approach that measures cleavage efficiency across a library of partially randomized nonanucleotide substrates resembling the canonical Rep recognition motif (5′ TATTATT*AC 3′). Substrates that remain uncleaved after reaction are selectively amplified and sequenced, allowing quantitative determination of cleavage frequency for each sequence variant by comparing to reference samples not treated with enzyme. This provides a comprehensive view of sequence specificity that correlates strongly with overall enzymatic activity in standard *in vitro* assays [20, 39]. We made several attempts to adopt HUHseq for RNA, but were unsuccessful. Our approach here is to benchmark sequence specificity of E1 and E2 compared to WT on DNA and then validate the findings with RNA oligos. We assayed the WT, E1, and E2 enzyme variants of PCV2 across two conditions: high Mn^2+^ (1 mM) and low Mn^2+^ (50 µM). Briefly, triplicate HUH reactions were run, followed by amplification of the library oligos and NGS. Percent reduction of each of the 16 384 sequence “k-mers” was calculated as described in the methods. Using the HUH-seq data generated, we plotted sequence logos of high efficiency substrates based on percent reduction calculations of k-mers in the sequence library with a threshold of 50% reduction or greater (Fig. 5A). Because our library contained every possible substrate encoded within a randomized 7-mer ssDNA sequence, the sequence logos are extensive representations of the specificity profile of a given HUH-tag in a specified condition. These logo plots are paired with histograms of k-mers binned by percent reduction to inform on how many substrates are being acted upon by the assayed enzyme and to what extent. The percent reduction scores used to generate these figures are mean values calculated across three replicates. Each condition had excellent correlation across replicates in read count per k-mer excluding WT PCV2 low Mn^2+^ replicate 1, which was excluded from mean value calculation (Supplementary Figs S7 and S8). The high metal condition for WT PCV2 closely resembles our previous HUH-seq results for this enzyme [20], showing a slight preference for its cognate substrate 5′ A_-7_A_-6_G_-5_T_-4_A_-3_T_-2_T_-1_*A_+1_C_+2_ 3′, and a high level of promiscuity (Fig. 5A). Surprisingly, the reduced metal condition only modestly improved the specificity of this enzyme even though it dramatically reduced the number of sequences the enzyme can efficiently cleave.

At high metal concentrations, both engineered HUH-tags exhibit pronounced promiscuity, showing little sequence preference across the substrate library and efficiently cleaving most of the 16 384 sequences (>50% reduction; Fig. 5A). This broad reactivity underscores a tradeoff between activity and specificity in the evolved enzymes. In contrast, under low Mn²⁺ conditions, sequence specificity is restored, with cleavage motifs resembling the canonical circovirus recognition sequence (5′ TATTATT*AC 3′).

Correlation analysis further supports this trend (Supplementary Fig. S8). Across all three enzymes, activity correlations between high and low metal conditions are weak, consistent with Mn²⁺-induced activation and loss of specificity. While WT activity correlates poorly with either engineered variant at high Mn²⁺ (r ≈ 0.5), E1 and E2 remain strongly correlated with each other (r = 0.91). Under low metal conditions, all three enzymes show improved agreement (E1 – E2, r = 0.82), reflecting a shared restoration of specificity. Together, these data indicate that the engineered variants maintain high activity while regaining sequence selectivity at reduced cation levels, making them well suited for RNA and DNA HUH-tag applications under physiologically relevant conditions.

To validate the HUHseq results and test whether HUH-seq results can be extrapolated to RNA substrates, we selected k-mer sequences from the HUH-seq dataset predicted to be cleaved by E2 with high, medium, or low efficiency (Fig. 5B). We then synthesized both DNA and RNA versions of these oligos and assayed their reactivity with the engineered E2 variant under high and low Mn²⁺ conditions (Fig. 5C and Supplementary Fig. S9). At high Mn²⁺ concentrations, both DNA and RNA oligos were broadly cleaved, reflecting the loss of specificity observed for engineered variants observed in HUHseq. By contrast, at low Mn²⁺ concentrations, cleavage efficiencies closely matched predictions from the HUH-seq data. These results not only confirm the increase in specificity at low metal concentrations but also demonstrate that DNA-based HUH-seq predictions can be used to make predictions about preferred RNA substrates.

### Comparative analysis of engineered PCV2 mutants

**Figure 6.**
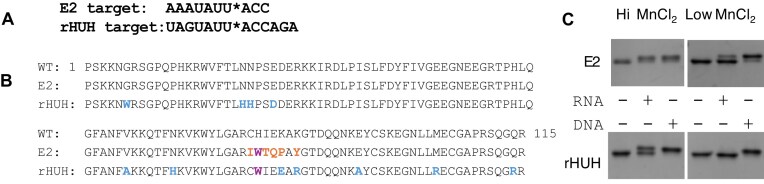
Comparison of engineered mutants across studies. (**A**) RNA sequences the E2 (this study) and rHUH [30] mutants were engineered to react with. (**B**) Protein sequences of engineered mutants compared to WT. Mutations compared to WT are colored orange and blue, and purple denotes the common mutation. (**C**) Reactivity of mutants with five-fold excess DNA and RNA after 10 min at high and low Mn²⁺.

Remarkably, the Ting lab also engineered RNA reactivity into PCV2 [30], enabling direct comparison of mutants. They performed seven rounds of directed evolution across the full PCV2 sequence, whereas we conducted two rounds focused on the sDBM DNA-binding region [20]. They also used a slightly different RNA substrate (Fig. 6A) and selected toward Mg^2+^ reactivity, while our selections were carried out in Mn²⁺. Their lead mutant, rHUH, contains 12 mutations compared to the 6 mutations in our E2 variant (Fig. 6B). Notably, both studies identified the H81W mutation, which was found to be the critical mutation in our study as previously discussed (Supplementary Fig. S5). While their study did not compare E2 to their rHUH in any *in vitro* assays where 1 mM Mn^2+^ was used as the divalent cation, they compared a yeast surface-displayed version of E2 to rHUH and found E2 to be less reactive than rHUH in the presence of 1 mM Mg^2+^. We sought to compare the mutants under Mn²⁺ conditions.

We expressed and purified rHUH alongside E2 using our standard protocols and assayed both under our laboratory’s HUH reaction conditions. With ∼5-fold excess nucleic acid, both enzymes reacted robustly with DNA and RNA at high Mn²⁺ concentrations in 10-min reactions (Fig. 6B). At low Mn²⁺ concentrations, however, differences emerged: E2 retained efficient RNA reactivity, whereas rHUH activity was markedly reduced (Fig. 6C and Supplementary Fig. S10).

Finally, we assayed specificity of the reaction by reacting E2 and rHUH with their respective target RNA oligos in the presence of background RNA. We used whole cell RNA extracted from S9 cells (Supplementary Fig. S11). As background RNA concentration increased, rHUH reactivity decreased, accompanied by loss of the expected protein–nucleic acid bands and the appearance of higher-molecular-weight smears, consistent with nonspecific association of the protein with RNA extracts, as previously noted in their manuscript. In contrast, the E2 variant retained strong activity even in the presence of background RNA, although a modest reduction in efficiency was observed at the highest RNA concentrations under low Mn²⁺ conditions.

## Discussion

HUH-tags have emerged as versatile tools for sequence-directed protein–ssDNA bioconjugation with applications ranging from imaging to genome editing [20, 22–29, 49, 50]. Here, we expand this platform by demonstrating that HUH-endonucleases can be engineered to form covalent linkages with RNA substrates through directed evolution. Using yeast surface display, we evolved PCV2 variants that exhibit robust RNA reactivity, maintaining high sequence specificity at low Mn²⁺ concentrations and retaining activity even in the presence of exogenous cellular RNA. These features position RNA-reactive HUH-tags as promising fusion partners for covalently tethering proteins to designer RNA sequences, enabling programmable colocalization of effector domains with transcripts of interest.

Our engineered mutants also displayed enhanced DNA activity, particularly under high metal conditions. This property could be harnessed in applications such as click editing [50], where HUH-tags tether donor DNA templates to genome editing enzymes. Increased DNA linkage efficiency within mammalian cells could improve substrate recruitment and editing outcomes, suggesting that the variants reported here may be directly useful for advancing this technology. Moreover, the ability to engineer HUH-tags with designer sequence specificity would dramatically improve the potential for multiplexing with this bioconjugation platform. While we have identified high efficiency noncross reactive substrates across pairs, triplets, and even quadruplets of HUH-tags, the vast majority of the sequence space encoded by their substrate length is completely inaccessible to these enzymes, particularly sequences enriched in G content [20, 39]. The results presented here lay the foundation of exciting new possibilities for expanding and fine-tuning the functionality of HUH-tags to suit an even broader range of applications in biotechnology and synthetic biology.

Comparison with the independently evolved rHUH mutant from the Ting lab [30] revealed both convergent and divergent solutions to RNA reactivity. Notably, both studies identified the H81W mutation as critical for enabling RNA activity, underscoring its importance across engineering strategies. Yet functional benchmarking revealed sharp contrasts: rHUH performed better than E2 in yeast display assays under Mg²⁺, whereas our biochemical assays under Mn²⁺ showed E2 retained robust RNA reactivity at low Mn²⁺, while rHUH activity sharply decreased. Moreover, rHUH lost activity in the presence of background RNA and formed nonspecific aggregates, whereas E2 maintained specificity under these conditions. These results suggest complementary strengths; rHUH’s improved performance under physiologic Mg²⁺ versus E2’s greater specificity and stability that could be combined in future engineering efforts.

Despite these advances, several challenges remain that currently limit the use of these enzymes in mammalian cells. First, the engineered variants exhibit reduced stability compared to the WT, reflecting the common trade-offs associated with directed evolution [31, 51]. Second, while the mutants display decreased sequence specificity, this effect is largely mitigated under low Mn²⁺ concentrations similar to those found in cells. However, although E2 retains specificity under these conditions, its catalytic efficiency at physiologically relevant Mn²⁺ levels remains lower than desirable for cellular applications. Future engineering efforts focused on enhancing protein stability and tuning metal ion dependence will be essential to fully realize the potential of RNA-reactive HUH-tags *in vivo*. Given the estimated Mn²⁺ dissociation constant of ∼90 μM, one promising strategy could involve engineering variants with higher Mn²⁺ affinity.

Covalent protein–RNA linkage is not unique to engineered systems and occurs naturally, most notably in viruses. In poliovirus and related RNA viruses, the 5′ end of the genome is covalently attached to a viral protein known as VPg [52] through a phosphotyrosine bond, similar in chemistry to the engineered HUH-tag described here, which primes RNA replication. Additionally, certain phage ADP-ribosyltransferases can use NAD-capped RNAs as substrates, covalently attaching RNA to host proteins in a process termed “RNAylation” [53]. Even translation involves a form of RNA linkage, through the covalent attachment of amino acids to their corresponding tRNAs. Intriguingly, the ability of Reps to interact promiscuously with RNA is intriguing in light of their evolutionary history. Rep proteins share structural similarity with viral RNA recognition motifs [14, 54], and Cruciviridae CRESS-DNA viruses thought to arise from recombination between ssDNA and RNA viruses encode Reps closely related to circoviruses [55–57]. The RNA reactivity uncovered here may therefore echo an ancestral capacity for bridging DNA and RNA viral replication mechanisms.

Together, these findings establish RNA-reactive HUH-tags as a compact and engineerable platform for sequence-directed protein–RNA linkage. Beyond enabling protein–RNA colocalization, their improved DNA reactivity also broadens their potential utility in genome engineering applications such as click-editing. At the same time, their current limitations underscore the need for further engineering to balance activity, specificity, stability, and metal dependence. More broadly, this work highlights the plasticity of HUH-endonucleases and sets the stage for tailoring their activity and specificity to meet the demands of emerging biotechnologies.

## Supplementary Material

gkag517_Supplemental_Files

## Data Availability

The custom python script used to analyze HUH-seq Next-Generation sequencing data is available on GitHub (https://github.com/AdamTSmiley) and Zenodo (https://doi.org/10.5281/zenodo.19819901) under an open source license. Processed Next-Generation sequencing data from the HUH-seq assay and both rounds of protein engineering are available as supplemental data files.
