# Supplementary material for: Sequence-directed covalent protein–RNA linkages in a single step using engineered HUH-tags
Source: Nucleic Acids Res. 2026 Jun 3;54(10):gkag517. doi: 10.1093/nar/gkag517 (PMC13231165; doi:10.1093/nar/gkag517)
Supplement: gkag517_Supplemental_Files [file gkag517_supplemental_files.zip › HUHRNA_SI_R3F.pdf]

Supplementary Information

Sequence-Directed Covalent Protein-RNA Linkages in a Single Step Using Engineered HUH-Tags

Adam T. Smiley*, Calvin J. Thoma, Natalia Babilonia-Díaz, August J. Krueger, Andrew C.D. Lemmex, Aspen J. Hughes, Matthew R. Pawlak, Kassidy J. Tompkins, Robert P. Connacher, Hideki Aihara, and Wendy R. Gordon*

Department of Biochemistry, Molecular Biology, and Biophysics, University of Minnesota, Minneapolis, Minnesota 55455, United States

*Correspondence: smile073@umn.edu & wrgordon@umn.edu

Supplemental Tables, Data, & Figures

Supplementary Table 1 - Enzymes/Abbreviations in Manuscript

HUH-tag	Viral Species/Full Name	Viral Family	Accession	Substrate
PCV2	Porcine circovirus 2	*Circoviridae*	NC_005148	AAGTATT*AC
DCV	Muscovy duck circovirus	*Circoviridae*	KR491947	TATTATT*AC
WDV	Wheat dwarf virus	*Geminiviridae*	AJ311031	TAATATT*AC
CoCV	Columbid Circovirus	*Circoviridae*	NC_002361	TAGTATT*AC
ePCV2 E1	Engineered PCV2 Variant 1	-	-	rArArArUrArUrU*rArC
ePCV2 E2	Engineered PCV2 Variant 2	-	-	rArArArUrArUrU*rArC
ePCV2 rHUH	Engineered PCV2 variant from Ting lab	-	-	rUrArGrUrArUrU*rArC

Supplementary Table 2 - Oligonucleotide Substrates

Name	Sequence	Figures
HUHRNA	rCrGrUrArArArArUrArUrU*rArCrCrGrUrC	1,3,6,S1, S2, S10
HUHDNA	CGTAAATATT*ACCGTC	1,3,6,S1, S2, S10
HUHDNA_Q/F	/5IABkFQ/CGTATATTATT*ACCGTC/36-FAM/	4
HUHRNA_Q/F	/5IABkFQ/rCrGrUrArArArArUrArUrU*rArCrCrGrUrC/36-FAM/	4
HUHRNA_FAM	rCrGrUrArArArArUrArUrU*rArCrCrGrUrC/36-FAM/	4,S3
HUHDNA_FAM	CGTAAATATT*ACCGTC/36-FAM/	4,S3
HUH_Seq	A*C*G*A*A*A*G*A*A*G*T*G*C*G*C*T*G*T*NNNNNNNACCC*T*T*T* T*G*G*A*A*T*T*C*T*C*G*G*G*T*G*C*C*A*A*G*G*C	5
HUHHyb_Bio	CGTArArArArUrArUrU*rArCrCGTC/3Bio/	2
HUHHyb_AF647	CGTArArArArUrArUrU*rArCrCGTC/3AlexF647N/	2
rHUH target	rUrArGrUrArUrU*rArCrCrArGrA	7, S

Supplementary Data 1 - Amino Acid Sequences for WT Enzymes in Manuscript

>PCV2
PSKKNGRSGPQPHKRWVFTLNNPSEDERKKIRDLPISLFDYFIVGEEGNEEGRTPHLQGFANFVKKQTFN
KVKWYLGARCHIEKAKGTDQQNKEYCSKEGNLLMECGAPRSQGQR

>DCV
MAKSGNYSYKRWVFTINNPTFEDYVHVLEFCTLDNCKFAIVGEEKGANGTPHLQGFLNLRSNARAAALEE
SLGGRAWLSRARGSDEDNEEYCAKESTYLRVGEPVSKGRSS

>WDV
MASSSTPRFRVYSKYLFLTYPQCTLEPQYALDSLRTLLNKYEPLYIAAVRELHEDGSPHLHVLVQNKLRA
SITNPNALNLRMDTSPFSIFHPNIQAAKDCNQVRDYITKEVDSDVNTAEWGTFVAVSTPGRKDRDAD

>CoCV
REATRRPPREAAAKRWCFTLNNPTEEEIKSLETWLVSDFHYAIVGKEVGEQGTPHLQGFVHLKQKKRLPQ
LKQLFKRAHWEKARGSDEDNEKYCSKEGNVLLTLGIPAKGNR

Supplementary Data 2 - Amino Acid Sequences for Engineered Enzymes in Manuscript

*Legend: **Bold** = Mutagenized; Black Font = Same as WT; Red Font = Different then WT*

>ePCV2_E1
PSKKNGRSGPQPHKRWVFTLNNPSEDERKKIRDLPISLFDYFIVGEEGNEEGRTPHLQGFANFVKKQTFN
KVKWYLGA**RVWLQP**AKGTDQQNKEYCSKEGNLLMECGAPRSQGQR

>ePCV2_E2
PSKKNGRSGPQPHKRWVFTLNNPSEDERKKIRDLPISLFDYFIVGEEGNEEGRTPHLQGFANFVKKQTFN
KVKWYLGAR**I**W**TQ**P**AY**GTDQQNKEYCSKEGNLLMECGAPRSQGQR

>ePCV2_rHUH (Ting lab)
PSKKNWRSGPQPHKRWVFTLHHPSDDERKKIRDLPISLFDYFIVGEEGNEEGRTPHLQGFANFAKKQTFH
KVKWYLGARCWIEEARGTDQQNKAYCSKEGNLLRECGAPRSQGRR

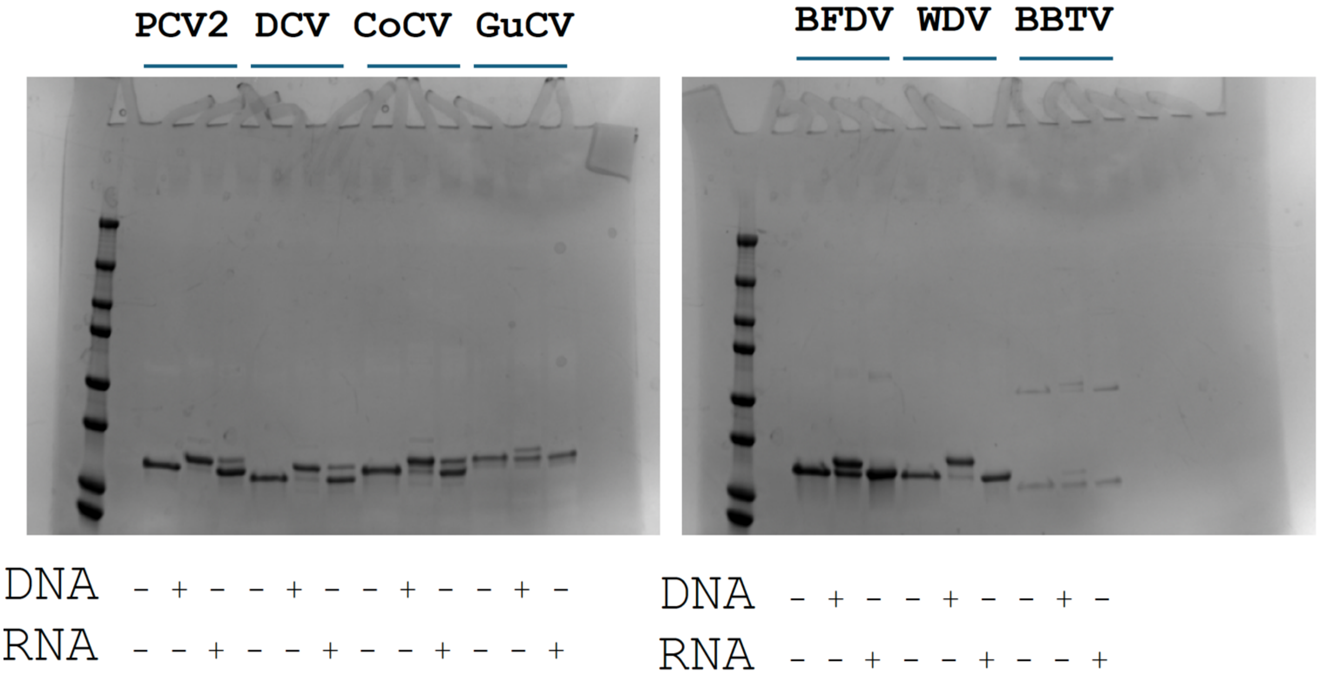

Supplementary Figure 1. Full-length gels for Figure 1B. Rep domains derived from circovirus and geminivirus HUH-endonucleases were expressed, purified, and reacted DNA/RNA oligos. These reactions were incubated overnight (16 hours) in the following concentrations: 3 μM HUH-tag, 30 μM nucleic acid substrate, 50 mM HEPES pH 8.0, 50 mM NaCl, 1 mM DTT, 1 mM $MnCl_2$ at 37°C.

DNA Reactions (WT, E1, E2) no DNA followed by 3 reactions with DNA for each quartet

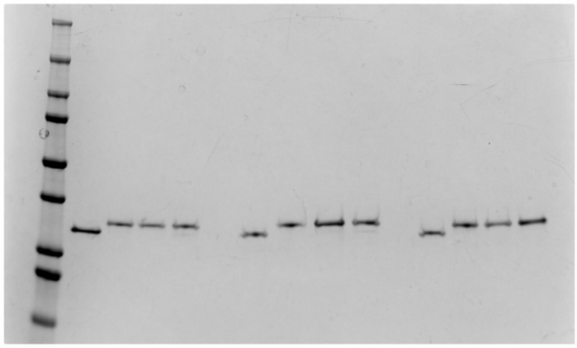

RNA Reactions (WT, E1, E2) no RNA followed by 3 reactions with RNA for each quarte

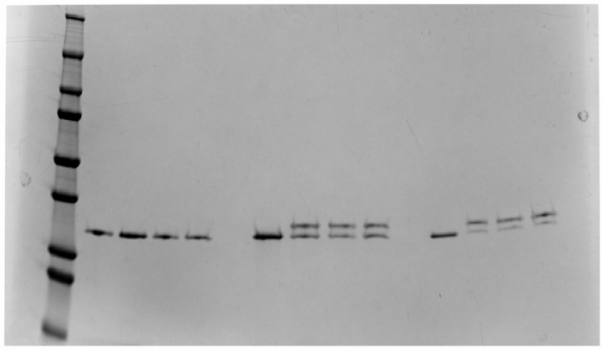

RNA Reactions for E2 at higher exposure for quantification no RNA followed by 3 reactions with RNA for each quartet

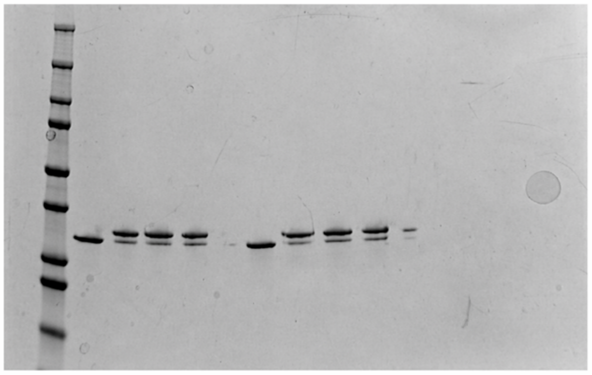

Supplementary Figure 2. Full gels for Figure 3. Replicates of PCV2 and engineered variants E1 and E2 reacted with DNA and RNA. Reactions were performed in final concentrations of 3 μM HUH-tag and 15 μM nucleic acid substrate in 50 mM HEPES pH 8.0, 50 mM NaCl, 1 mM DTT, and 50 μM MnCl2 for thirty minutes at 37°C. The second RNA reactions gel loaded more of the reaction to enable more accurate quantification of reaction efficiency for E2.

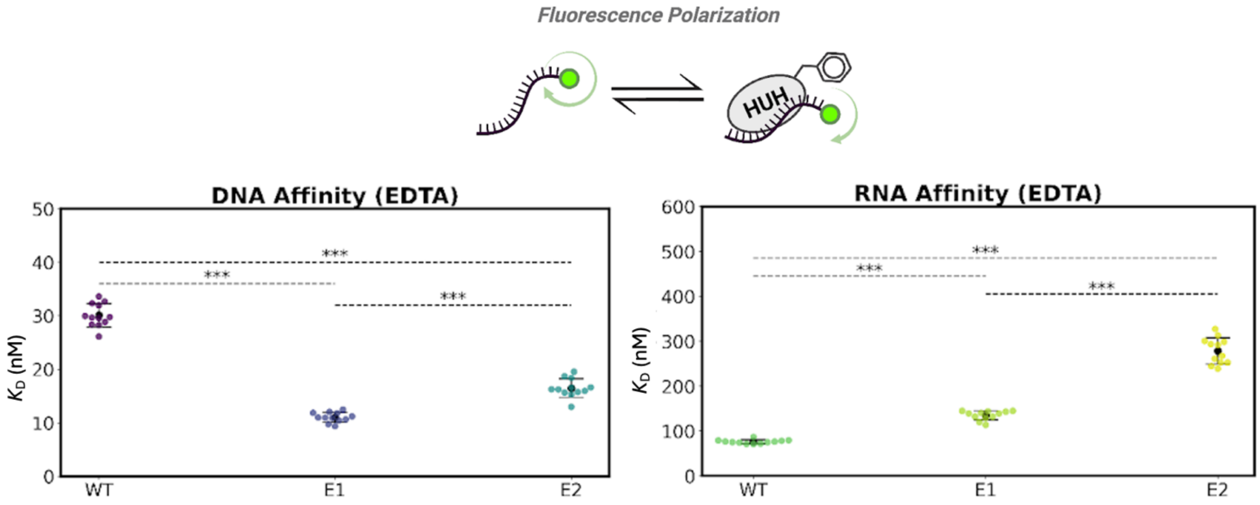

Supplementary Figure 3A: Graphical overview of the fluorescence polarization assay to gauge binding affinity, and the results of this assay with the WT, E1, and E2 variants on DNA (purple/blue color scheme) and RNA (green/yellow color scheme) substrates in absence of a catalytically required divalent cation. Data are presented as mean ± standard deviation. Statistical significance was determined using one-way ANOVA with Tukey’s multiple comparisons test (GraphPad Prism). n = 12 biological replicates. *** p<0.001.

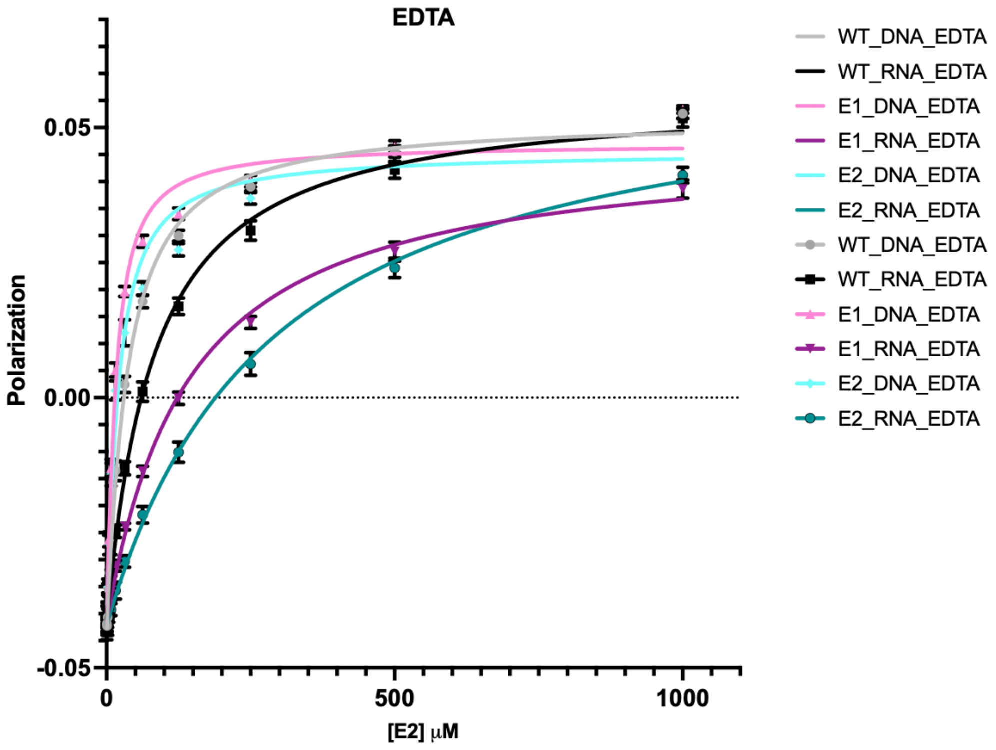

	WT_DNA_EDTA	WT_RNA_EDTA	E1_DNA_EDTA	E1_RNA_EDTA	E2_DNA_EDTA	E2_RNA_EDTA
FP [9]						
Best-fit values						
Amax	-0.0435600	-0.0412900	-0.041580	-0.0413900	-0.039530	-0.0418800
Amin	-0.0438000	-0.0415300	-0.041800	-0.0416200	-0.039750	-0.0421400
L	10.0000000	10.0000000	10.000000	10.0000000	10.000000	10.0000000
Kd	30.0300000	75.9600000	10.970000	134.6000000	16.430000	276.4000000
95% CI (profile likelihood)						
Amax	-0.0442200	-0.0418100	-0.042760	-0.0418500	-0.041240	-0.0423300
Amin	-0.0444600	-0.0420600	-0.042990	-0.0420700	-0.041450	-0.0425900
Kd	28.5300000	72.4900000	9.932000	127.7000000	14.080000	260.5000000
Goodness of Fit						
Degrees of Freedom	141.0000000	141.0000000	141.000000	141.0000000	141.000000	141.0000000
R squared	0.9966000	0.9970000	0.990300	0.9963000	0.978200	0.9958000
Sum of Squares	0.0006103	0.0004835	0.001601	0.0004164	0.003317	0.0004664
Sy.x	0.0020810	0.0018520	0.003370	0.0017190	0.004850	0.0018190
Constraints						
L	10.0000000	10.0000000	10.000000	10.0000000	10.000000	10.0000000
Kd	0.0000000	0.0000000	0.000000	0.0000000	0.000000	0.0000000
Number of points						
# of X values	144.0000000	144.0000000	144.000000	144.0000000	144.000000	144.0000000
# Y values analyzed	144.0000000	144.0000000	144.000000	144.0000000	144.000000	144.0000000

Supplementary Figure 3B: Raw data for fluorescence polarization figures- EDTA. Polarization was measured as a function of enzyme concentration in the presence of 1mM EDTA for WT, E1 and E2 with DNA and RNA targets. 12 measurements over 3 days were measured and fit to the equation in the methods using Graphpad Prism, and K_D was extracted. Error bars show standard deviation.

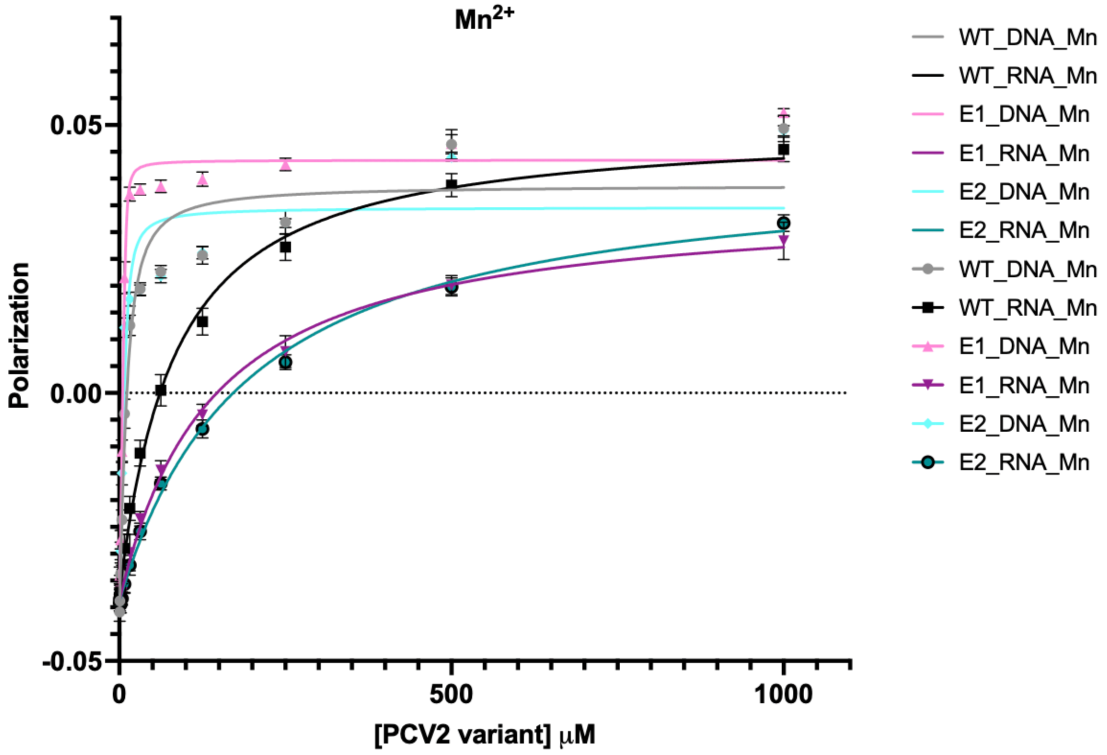

	WT_DNA_Mn	WT_RNA_Mn	E1_DNA_Mn	E1_RNA_Mn	E2_DNA_Mn	E2_RNA_Mn
FP [9]						
Best-fit values						
Amax	-0.042060	-0.037480	-0.041070	-0.0386400	-0.040410	-0.0387700
Amin	-0.042260	-0.037700	-0.041280	-0.0388300	-0.040600	-0.0389700
L	10.000000	10.000000	10.000000	10.0000000	10.000000	10.0000000
Kd	5.662000	75.390000	0.360500	131.0000000	1.835000	184.3000000
95% CI (profile likelihood)						
Amax	-0.044480	-0.038250	-0.042540	-0.0393200	-0.043380	-0.0392500
Amin	-0.044690	-0.038470	-0.042750	-0.0395100	-0.043580	-0.0394500
Kd	4.350000	69.810000	0.203300	119.3000000	1.032000	172.1000000
Goodness of Fit						
Degrees of Freedom	141.000000	141.000000	141.000000	141.0000000	141.000000	141.0000000
R squared	0.958700	0.992200	0.984400	0.9885000	0.931200	0.9940000
Sum of Squares	0.005879	0.001019	0.002394	0.0009183	0.008683	0.0004955
Sy.x	0.006457	0.002688	0.004120	0.0025520	0.007848	0.0018750
Constraints						
L	10.000000	10.000000	10.000000	10.0000000	10.000000	10.0000000
Kd	0.000000	0.000000	0.000000	0.0000000	0.000000	0.0000000
Number of points						
# of X values	144.000000	144.000000	144.000000	144.0000000	144.000000	144.0000000
# Y values analyzed	144.000000	144.000000	144.000000	144.0000000	144.000000	144.0000000

Supplementary Figure 3C: Raw data for fluorescence polarization figures- Mn^{2+}. Polarization was measured as a function of enzyme concentration in the presence of 50μM Mn^{2+} for WT, E1 and E2 with DNA and RNA targets. 12 measurements over 3 days were measured and fit to the equation in the methods, and K_D was extracted. Error bars show standard deviation.

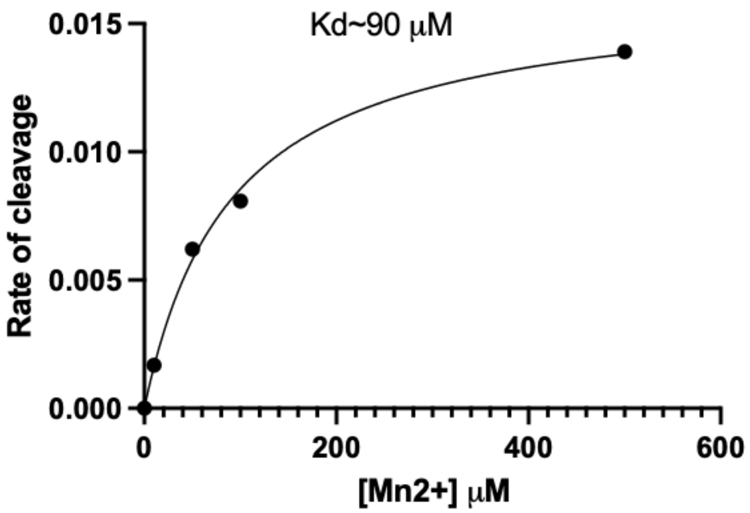

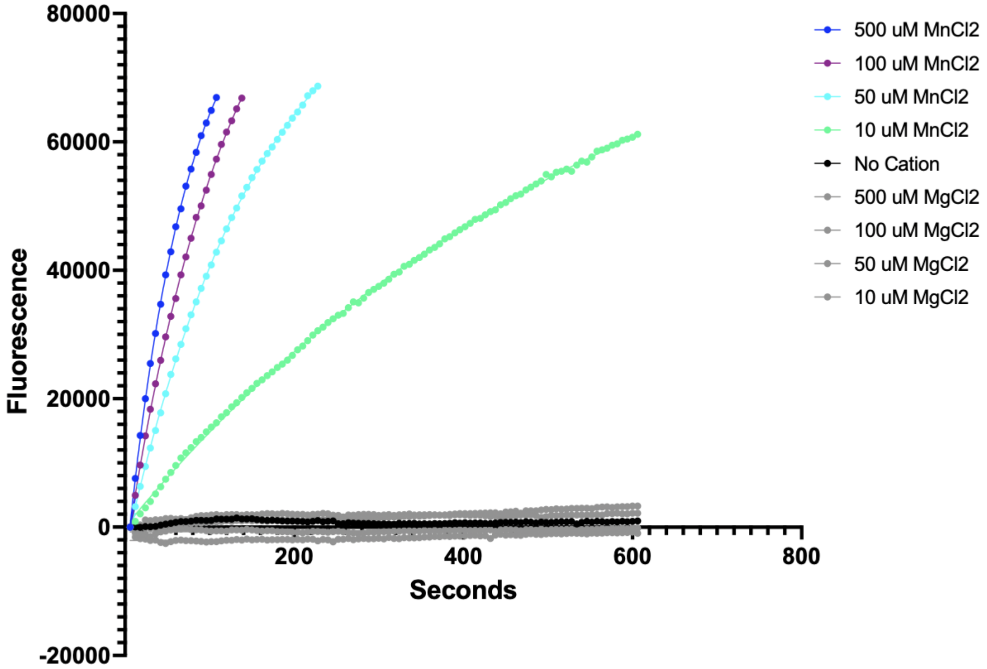

Supplementary Figure 4. Mg^{2+} and Mn^{2+} effects on E2 cleavage of RNA substrate. Metals were titrated into 1μM E2 and 100μM RNA beacon. Under these conditions. Mg^{2+} did not catalyze the cleavage reaction. Initial rates were fit and plotted to estimate the $K_{D.}$

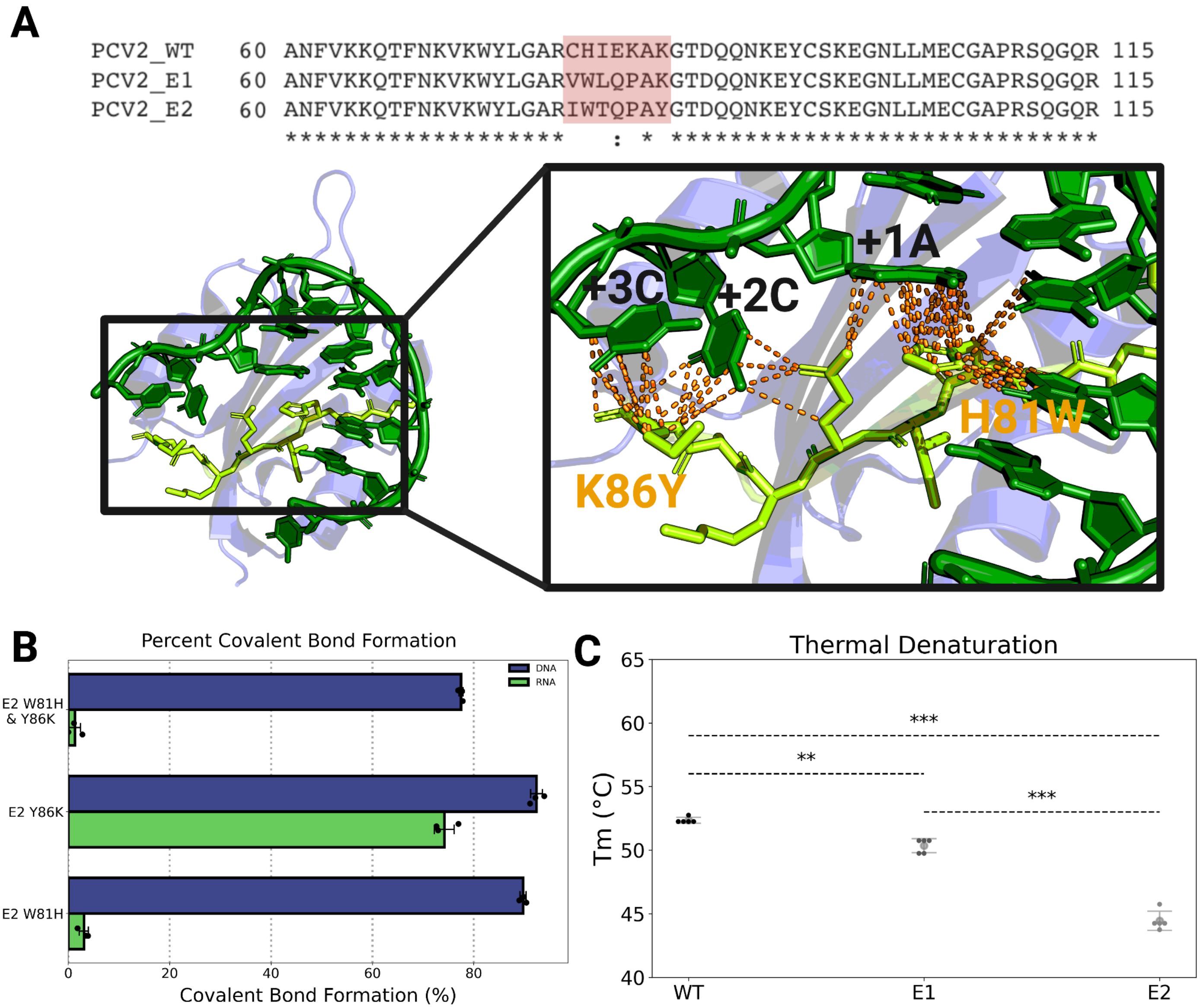

Supplementary Figure 5: Analysis of key mutations. (A) Partial sequence alignment showing the differences between the wildtype and engineered variants above a co-crystal structure of PCV2 in complex with its cognate DNA substrate. The image of the structure highlights the single-stranded DNA bridging motif, which was the primary target of engineering efforts. (B) *in vitro* HUH-tag bioconjugation reactions visualized via SDS-PAGE in triplicate represented as bar plots for both DNA (blue) and RNA (green) substrates with the indicated modified enzyme variants. Reactions were performed in final concentrations of 3 µM HUH-tag and 15 µM substrate in 50 mM HEPES pH 8.0, 50 mM NaCl, 1 mM DTT, and 50 µM MnCl2 for thirty minutes at 37°C. Error bars show standard deviations. (C) Strip plot demonstrating the differences in T_m as calculated by differential scanning fluorimetry (DSF) after each step of engineering. Error bars show standard deviation. ***p<0.001; **p<0.01

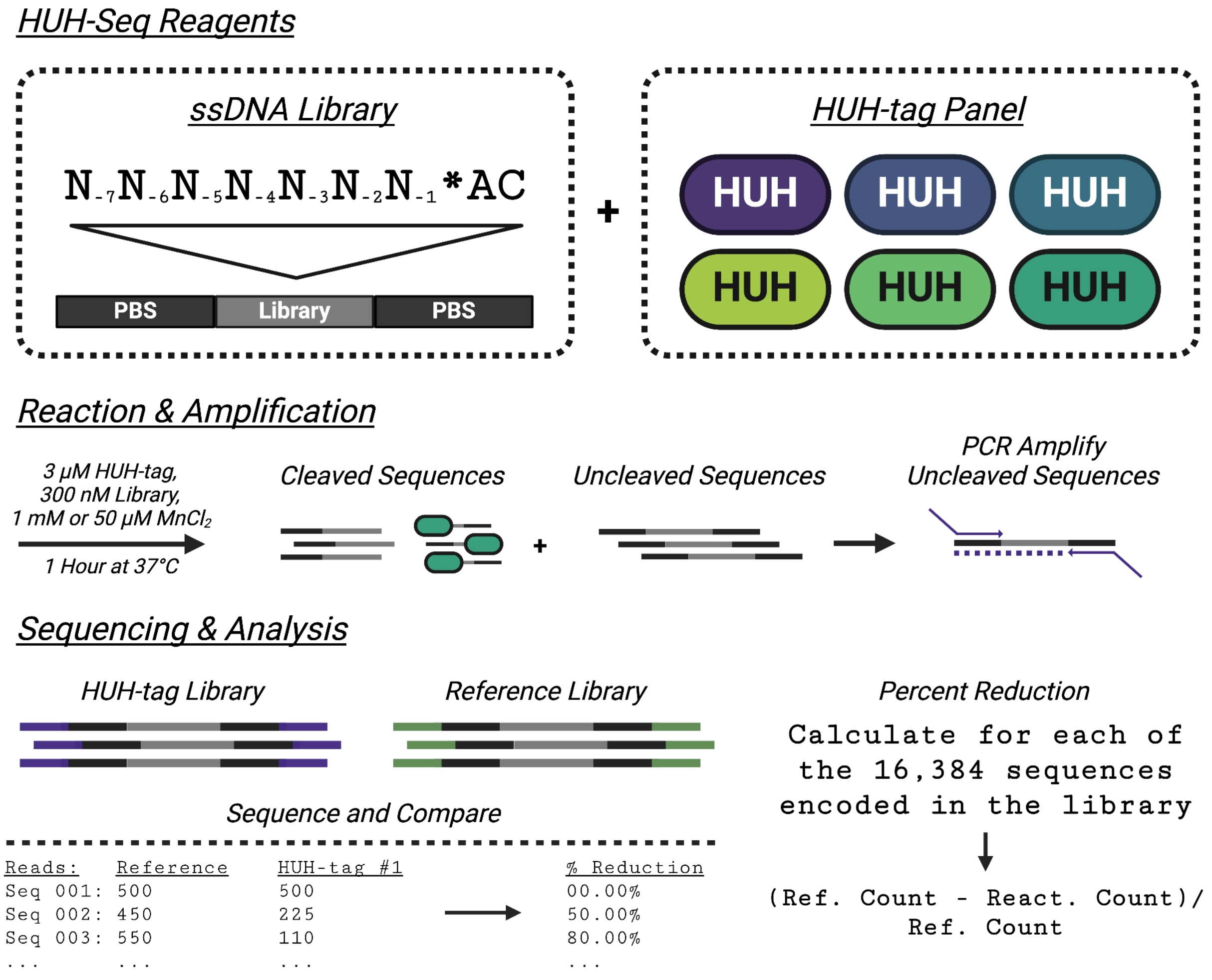

Supplementary Figure 6: HUH-Seq Graphical Overview. Graphical overview of HUH-seq, an NGS-based method for evaluating the cleavage specificity profiles of rep type HUH-tags. A degenerate ssDNA library containing seven randomized bases (4 bases ^ 7 positions = 16,384 unique sequences, or ‘k-mers’) flanked by primer binding sites (PBS) is reacted with a panel of HUH-tags, or no enzyme as a reference, in triplicate, generating two populations – the ‘Cleaved Sequences’ that were acted upon by an HUH-tag and the ‘Uncleaved Sequences’ which are the intact ssDNA library. A subsequent PCR amplification will amplify exclusively the uncleaved sequences (because they have PBSs that are still physically connected) and append next-generation sequencing adapters to their 5’ and 3’ ends. Each set of amplicons are then barcoded, pooled, and deep sequenced. A custom python script generates read counts for each k-mer across each reaction and subsequently calculates cleavage extent by a given HUH-tag per k-mer as percent reduction with respect to the reference library.

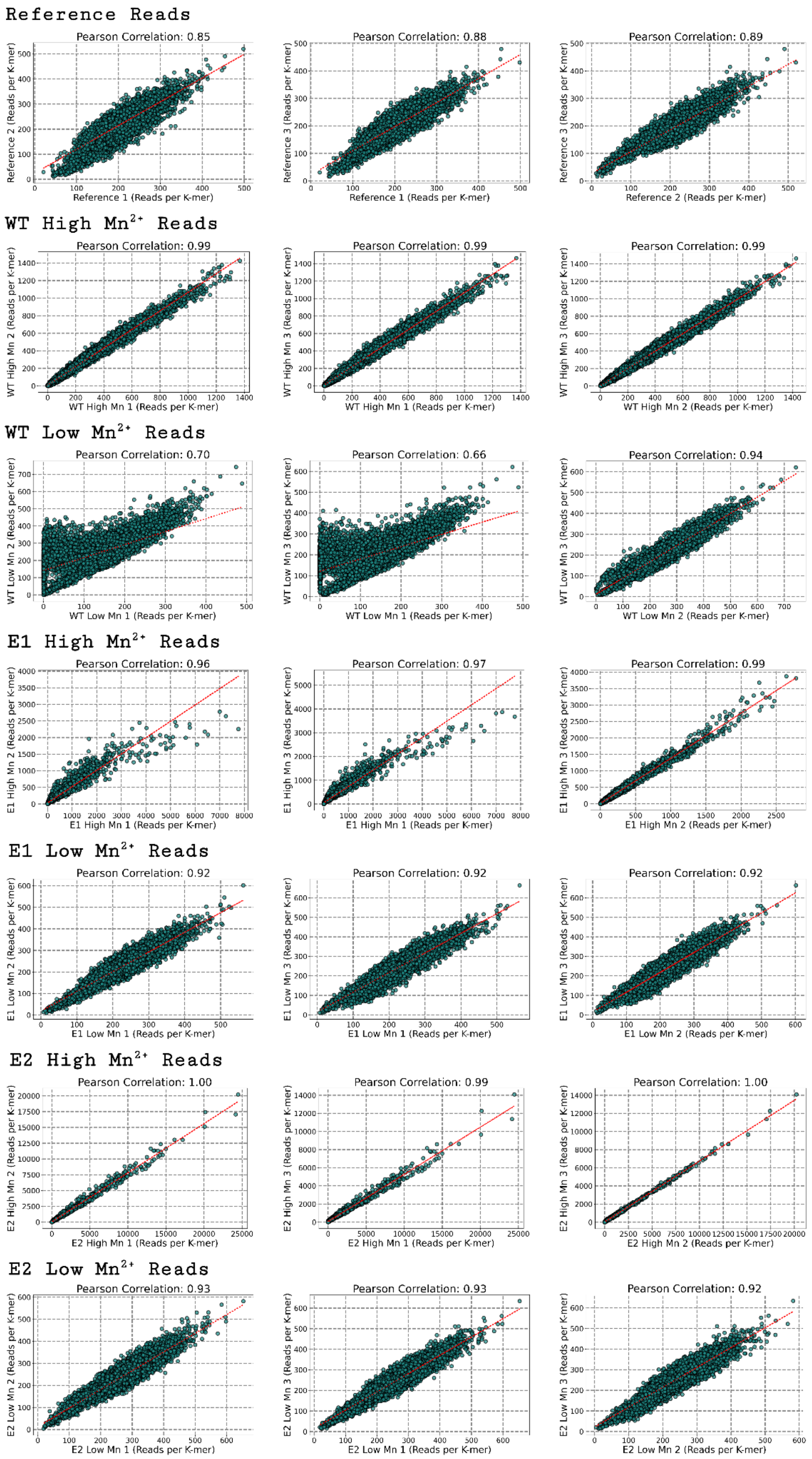

Supplementary Figure 7: Scatterplots showing the correlation between HUH-seq read counts per unique k-mer across three replicates in each of the seven specified conditions.

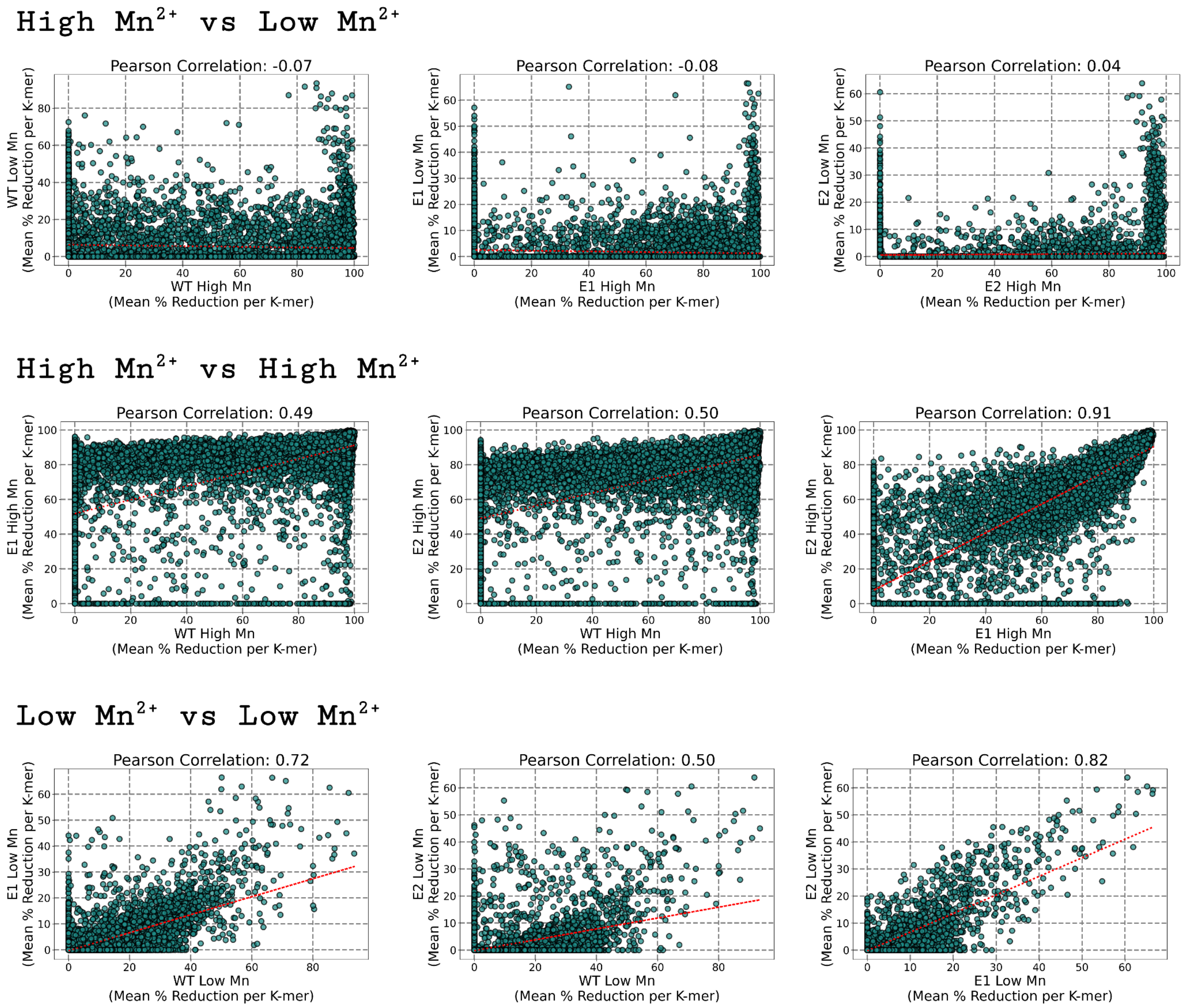

Supplementary Figure 8: Scatterplots showing the correlation between HUH-seq mean percent reduction per unique k-mer across the specified variants and conditions.

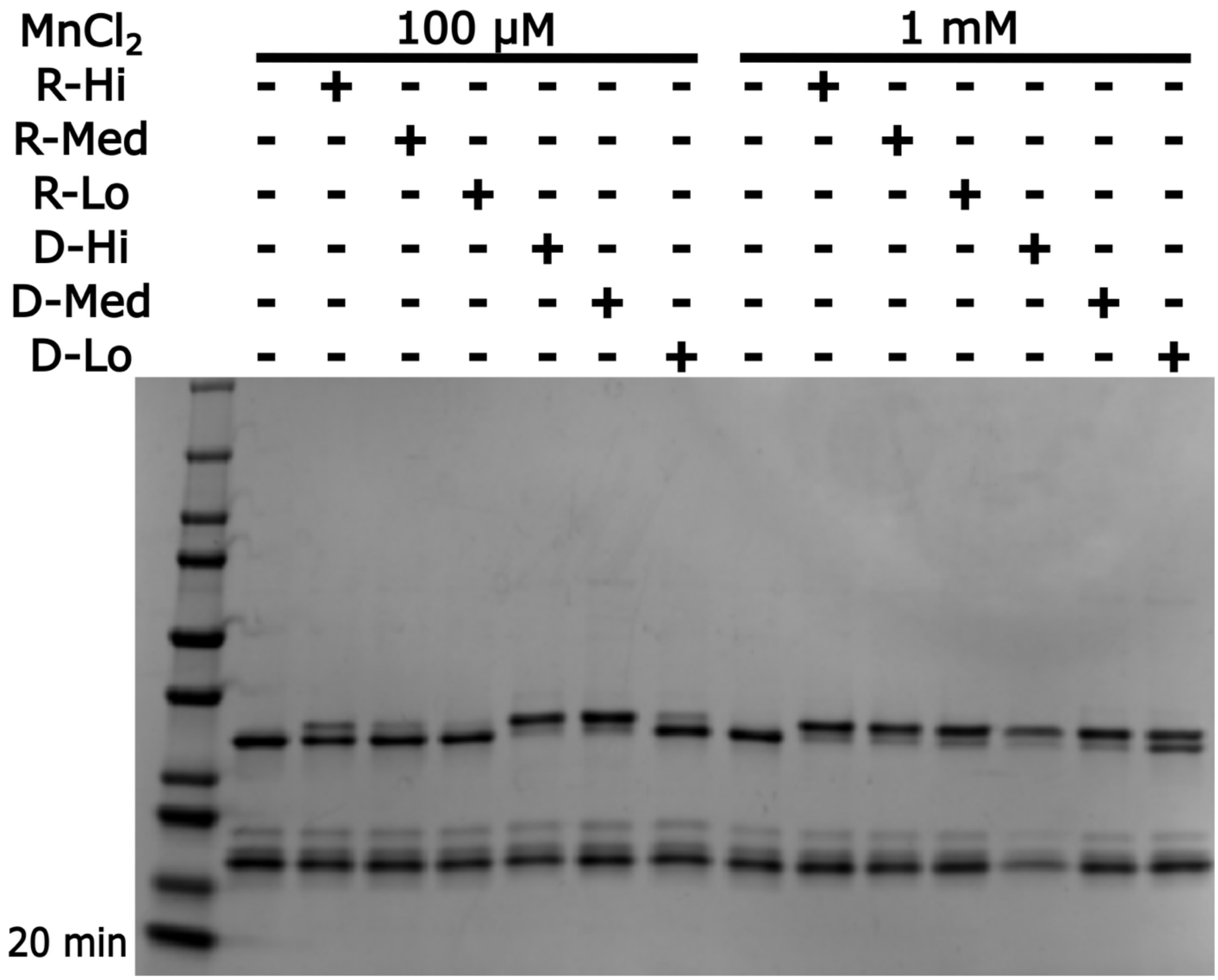

Supplementary Figure 9: Full gel for Figure 5.

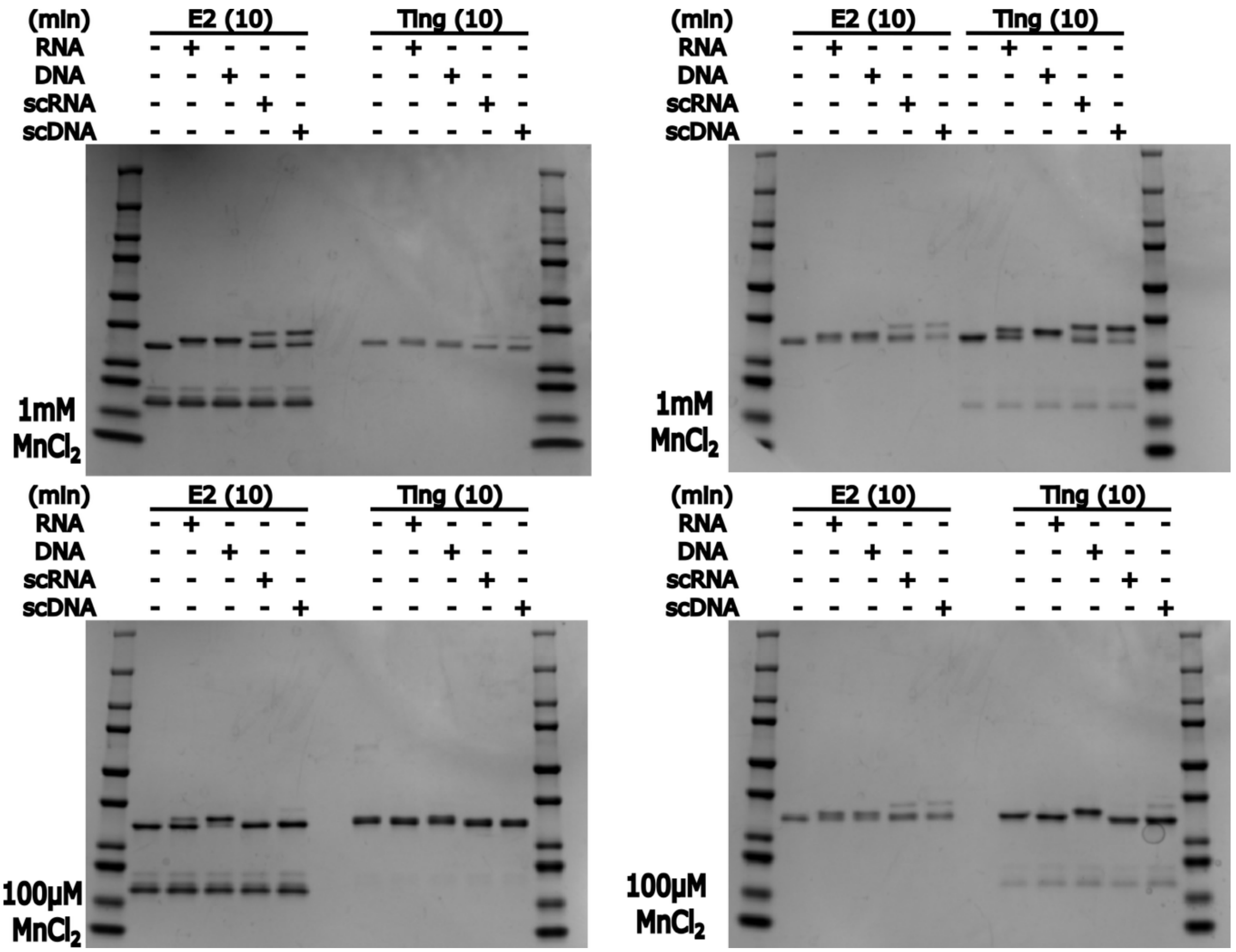

Supplementary Figure 10: Full gels for Figure 6C.

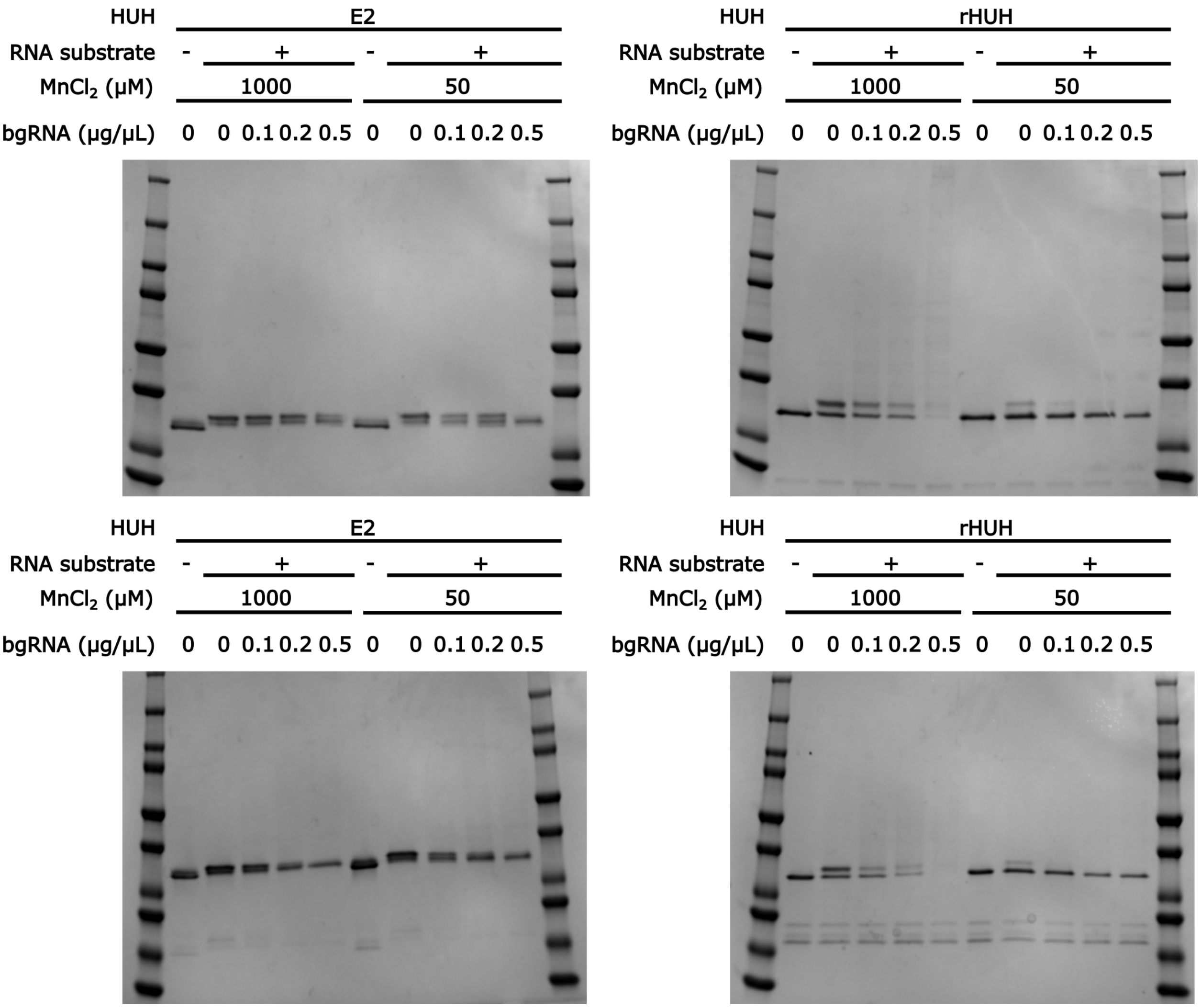

Supplementary Figure 11: Full gels for Figure 6D. E2 and rHUH were reacted with their respective target RNA sequences in the presence of RNA extracts from S2 cells.
